# VE-Cadherin Cleavage by LasB Protease from *Pseudomonas aeruginosa* Facilitates Type III Secretion System Toxicity in Endothelial Cells

**DOI:** 10.1371/journal.ppat.1003939

**Published:** 2014-03-13

**Authors:** Guillaume Golovkine, Eric Faudry, Stéphanie Bouillot, Romé Voulhoux, Ina Attrée, Philippe Huber

**Affiliations:** 1 INSERM, U1036, Biology of Cancer and Infection, Grenoble, France; 2 CNRS, ERL 5261, Bacterial Pathogenesis and Cellular Responses, Grenoble, France; 3 Université Joseph Fourier-Grenoble I, Grenoble, France; 4 CEA, DSV/iRTSV, Grenoble, France; 5 CNRS and Aix-Marseille Univ, Laboratoire d'Ingénierie des Systèmes Macromoléculaires (UMR7255), Marseille, France; Yale University, United States of America

## Abstract

Infection of the vascular system by *Pseudomonas aeruginosa* (*Pa*) occurs during bacterial dissemination in the body or in blood-borne infections. Type 3 secretion system (T3SS) toxins from *Pa* induce a massive retraction when injected into endothelial cells. Here, we addressed the role of type 2 secretion system (T2SS) effectors in this process. Mutants with an inactive T2SS were much less effective than wild-type strains at inducing cell retraction. Furthermore, secretomes from wild-typeswere sufficient to trigger cell-cell junction opening when applied to cells, while T2SS-inactivated mutants had minimal activity. Intoxication was associated with decreased levels of vascular endothelial (VE)-cadherin, a homophilic adhesive protein located at endothelial cell-cell junctions. During the process, the protein was cleaved in the middle of its extracellular domain (positions 335 and 349). VE-cadherin attrition was T3SS-independent but T2SS-dependent. Interestingly, the epithelial (E)-cadherin was unaffected by T2SS effectors, indicating that this mechanism is specific to endothelial cells. We showed that one of the T2SS effectors, the protease LasB, directly affected VE-cadherin proteolysis, hence promoting cell-cell junction disruption. Furthermore, mouse infection with *Pa* to induce acute pneumonia lead to significant decreases in lung VE-cadherin levels, whereas the decrease was minimal with T2SS-inactivated or LasB-deleted mutant strains. We conclude that the T2SS plays a pivotal role during *Pa* infection of the vascular system by breaching the endothelial barrier, and propose a model in which the T2SS and the T3SS cooperate to intoxicate endothelial cells.

## Author Summary

*Pseudomonas aeruginosa* (*Pa*) is a leading agent of nosocomial infections in humans, and clinical isolates are often multiresistant to antibiotics. As with most Gram-negative bacteria, *Pa* possesses a type III secretion system which consists of an injectisome through which the bacterium injects exotoxins inducing cytoskeleton collapse and apoptosis. *Pa* also delivers various toxins in the extracellular milieu by the type II secretion system, including the protease LasB. In order to disseminate throughout the body from the infection site and eventually reach the blood, the bacterium generally needs to cross the main barriers of the organism: the epithelium, the basal lamina and the vascular endothelium. Here we show that LasB specifically cleaves one main component of endothelial cell-to-cell junctions, the adhesive protein VE-cadherin, thus leading to junction disruption and endothelial barrier breakdown. VE-cadherin proteolysis also facilitates the action of type III exotoxins in endothelial cells. This cleavage mechanism is likely of major importance in *Pa* pathogenesis, as suggested by our bacterial dissemination experiments in mice.

## Introduction

*Pseudomonas aeruginosa* is an opportunistic Gram-negative pathogen that is responsible for nosocomial infections. It can cause chronic infections, which especially afflict cystic fibrosis patients, and acute infections, which often occur in patients bearing internal medical devices like ventilators, blood and urine catheters, or injection locks. *P. aeruginosa*-infected patients are difficult to cure because clinical strains are often multiresistant to antibiotics. Mortality rate was estimated to be 20–75% in *P. aeruginosa*-infected patients depending on infection site and antibiotic treatments (see [1], [2] and references therein).

Like most Gram-negative bacteria, *P. aeruginosa* utilizes several virulence factors during the infection process. The mortality of model animals was attributed to two major virulence determinants, the type 3 secretion system (T3SS) and the type 2 secretion system (T2SS) [3], [4]. The T3SS consists of an injectisome that is synthesized and assembled on the bacterial surface once bacteria are in the vicinity of host cells [5]–[7]. This syringe-like system injects toxins directly into the cytoplasm of target cells. Four T3 exotoxins have been identified in *P. aeruginosa* strains: ExoS, ExoT, ExoU and ExoY. ExoS and ExoT are highly homologous bifunctional toxins endowed with both GTPase-activating-protein (GAP) and ADP-ribosyl-transferase (ADPRT) activities. ExoS and ExoT target and inactivate various substrates, including GTPases and adaptor proteins. This ultimately produces cofilin activation, actin cytoskeleton dismantlement, focal adhesion loss and cell apoptosis [7], [8]. ExoU has very efficient phospholipase activity that permeabilizes the plasma membrane and rapidly leads to necrotic cell death [7]. ExoY is an adenylate cyclase, whose toxicity remains elusive [8], [9]. *P. aeruginosa* strains usually secrete a maximum of 3 exotoxins, ExoS and ExoU being mutually exclusive. The T3SS is required for toxicity and bacterial dissemination in mouse models of acute pneumonia [4]. Furthermore, the T3SS was shown to be necessary for bacterial survival in the blood in septicemic models [4].

Another secretion system contributing to *P. aeruginosa* virulence is the T2SS [10], [11]. This system secretes several proteases into the extracellular milieu, including elastases LasA and LasB, protease IV (PrpL) and IMPa (PmpA), as well as phospholipases and a potent toxin named exotoxin A. Type 2 secretion is a two-step process: effectors first translocate in the periplasm using Sec or Tat export machinery and are subsequently ejected into the surrounding medium by a piston-like structure called the type 2 secretion [12]. LasB is the major extracellular protease that degrades matrix proteins such as elastin, fibronectin and vitronectin, as well as a restricted number of cell receptors [10], [11]. The protease IMPa is an immunomodulating metalloprotease that prevents neutrophil extravasation and thereby protects *P. aeruginosa* from neutrophil attack [13]. Type 2 effectors are thought to be major agents of basal lamina destruction and immunomodulation. T2SS toxicity was recently examined using a mouse acute pneumonia model [3]. In absence of the T3SS, the T2SS-positive strain induced mouse death, albeit delayed compared to the T3SS-positive strain. Bacteria lacking both secretion systems were avirulent. This result tends to demonstrate that the T2SS contributes to bacterial pathogenicity, although to a lesser extent than T3SS effectors.

In this work we explored the effects of the T2SS of *P. aeruginosa* on endothelial cells. These cells are exposed to bacteria either through blood-borne infections or when bacteria disseminate from mucosa infection primary sites. In the latter situation, systemic infection is achieved when bacteria cross the epithelial or mesothelial barrier, the basal lamina and the endothelium.

In endothelial cells, we recently reported that the T3SS induces striking cell retraction that parallels increased paracellular permeability [8]. Here, we show that T2SS inactivation delayed T3SS-induced cell retraction. *P. aeruginosa*'s secretomes induced gap formation between cells only when strains were T2SS competent. We show that LasB is required for this activity by directly cleaving VE (vascular endothelial)-cadherin, an essential adhesive component of endothelial adherens junctions, but LasB has no effect on epithelial (E)-cadherin. VE-cadherin proteolysis was also observed in mouse models of infection with *P. aeruginosa*. LasB-mediated cleavage releases the adhesive domain of VE-cadherin, rendering the protein unable to maintain cell-cell junctions. Based on these findings, a cooperation model is proposed between the T2SS and T3SS.

## Results

### *P. aeruginosa*'s T2SS contributes to rapid T3SS-dependent cell intoxication

**Figure 1:**
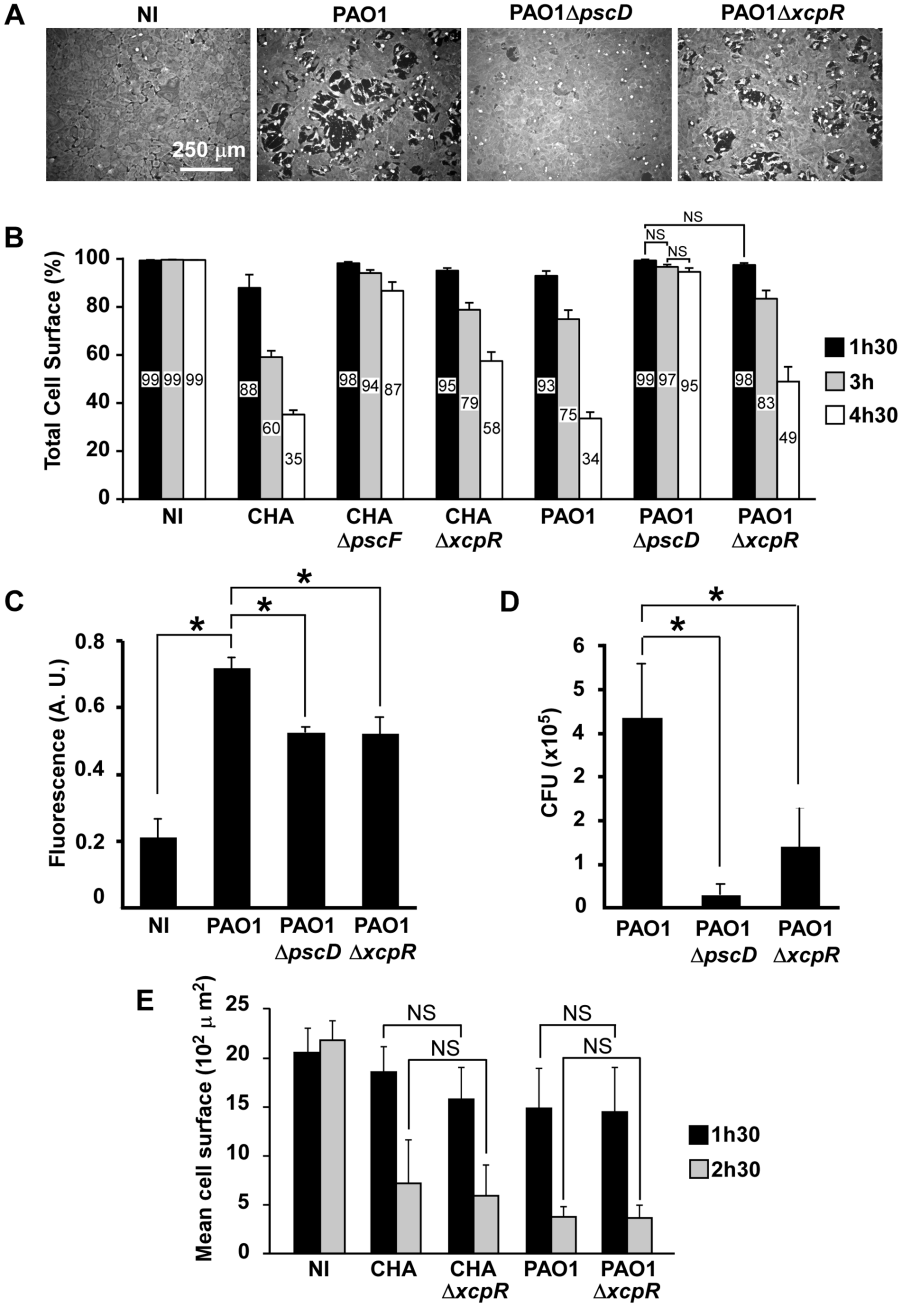
T3SS-induced cell retraction is influenced by T2SS. (A) Confluent HUVECs were treated for 3 h with PAO1, PAO1Δ*pscD* or PAO1Δ*xcpR* at MOI = 10. Control was non-infected cells (NI). Cells were fixed, actin labeled to stain the entire cell body and observed at low magnification. Images show major area of cell retraction when infected with PAO1, whereas only little retraction was observed with PAO1Δ*xcpR* and minimal retraction with PAO1Δ*pscD*. (B) Analogous experiment with strains CHA, CHAΔ*pscF*, CHAΔ*xcpR*, PAO1, PAO1Δ*pscD* and PAO1Δ*xcpR*. Cells were fixed at 1.5 h, 3 h and 4.5 h p.i.. For each image, the surface percentage occupied by cells in the field was measured by ImageJ software and presented as the mean percentage+SD (n = 10). The exact mean percentage is also indicated in the bars. Statistical analysis was performed using 2-way ANOVA for CHA and PAO1 data independently: p<0.001 for differences between wild-type/mutants and between times. Holm-Sidak's pairwise post-hoc test indicates that differences between wild-type/mutants at a given time, and between times for a given strain, are all significant, unless indicated (NS). (C) Confluent HUVECs cultured on porous membranes were infected at MOI of 10 with either PAO1, PAO1Δ*xcpR* or PAO1Δ*pscD*. Controls were uninfected cells (NI). Albumin-TRITC was simultaneously loaded in the upper compartments and TRITC fluorescence was measured at 7 hours p.i. Data are presented as mean fluorescent units (n = 3)+SD. Statistical differences were established with 1-way ANOVA (p<0.001), followed by Dunnett's test: all differences were significant when compared to PAO1 (*). (D) In the same experiment, bacterial transmigration was evaluated by CFU counts in the lower compartment at 7 hours p.i.. Data are presented as mean total CFU (n = 3)+SEM. Statistical differences were established as in (C): 1-way ANOVA: p = 0.019. (E) Cell retraction test on sparse HUVECs infected by bacterial strains. Data represent the total cell surface divided by the cell number in the field. Data are the mean percentage (n = 10)+SD. Statistical analysis: 2-way ANOVA for CHA and PAO1 independently (p<0.001, for differences between times and between wild-type/mutants). Holm-Sidak's pairwise post-hoc test indicates that differences between wild-type/mutants at a given time is not significant (NS).

As we previously reported, endothelial cell retraction by *P. aeruginosa* is dependent upon its T3SS [8]. Only small amounts of retraction were observed in absence of all T3SS effectors or the injectisome. Here, we wondered whether the T2SS contributes to T3SS-induced cell retraction. To this end, we used two *P. aeruginosa* strains, PAO1Δ*xcpR* and CHAΔ*xcpR*, both possessing a fully active T3SS (Fig. S1A), but lacking XcpR, a protein essential for secretion through the T2S pathway (Table 1) [14]. Infections were performed on HUVECs, which are primary cells derived from human umbilical cord veins (Fig. 1A,B). The lack of a functional T2SS in CHA and PAO1 resulted in significantly lower retraction when compared to the cognate wild-type strains at all times post-infection (p.i.). As controls, we used mutant strains lacking a functional T3SS (PAO1Δ*pscD* and CHAΔ*pscF*). Both T3SS-inactive strains induced minimal but significant cell retractions at 4.5 h.p.i. (Fig. 1A,B), thus confirming that the T3SS had the major toxic effect. The fact that T3SS-inactive strains still induced a low but significant retraction at 3 (for CHA) and 4.5 (for PAO1) h.p.i. compared to uninfected cells indicates that other factors, possibly those secreted by the T2SS, induced retraction. The extent of cell retraction induced by wild-type strains was higher than the sum of the retractions induced by the mutants lacking the T2SS and the T3SS at all times p.i. for the two strains, which is consistent with a synergistic rather than additive effect of the two secretion systems.

**Table 1:** *P. aeruginosa* strains used in this work.

Strains	Description	Refs
PAO1	Wild-type PAO1	ATCC 15692
PAO1Δ*xcpR*	Deletion of the ATPase gene of Xcp secretion system (T2SS)	This study
PAO1Δ*lasB*	Deletion of elastase (LasB) gene	[40]
PAO1F (RP1831)	Subclone of PAO1	[41]
PAO1FΔ*pscD* (RP1871)	Deletion of inner membrane component of T3SS	[41]
PAO1FΔ*3Tox* (RP1949)	Deletion of *exoS*, *exoT*, *exoY* genes	[41]
CHA	Cystic fibrosis clinical isolate	[21]
CHAΔ*xcpR*	Deletion of the ATPase gene of Xcp secretion system (T2SS)	This study
CHAΔ*pscF*	Absence of the needle protein PscF	[42]

To further establish the effect of the T2SS on cell retraction, we analyzed endothelial cell monolayer integrity during infection in a permeability assay using Transwell plates and albumin-TRITC as a tracer (Fig. 1C). Bacteria lacking either the T2SS or the T3SS induced smaller increases in tracer translocation than the wild-type strain bacteria. In the same experiments, bacterial transmigration across the monolayer was investigated by counting colony forming units (CFU) in the lower compartment of the Transwell plate (Fig. 1D). Again, bacteria lacking either the T2SS or the T3SS transmigrated less than the wild-type, although this difference was only significant for the strain devoid of its T3SS because of data dispersion. Taken together, these results indicate that the T2SS contributes to the cell retraction phenomenon and may be a helper in T3SS action and toxicity. Interestingly, the monolayer permeability assay was more sensitive to the effect of the T2SS than the cell retraction assay, suggesting that a small retraction is sufficient to disrupt the endothelial barrier for small tracers or even bacteria.

From the above data we hypothesized that T2SS effectors facilitate the action of the T3SS by provoking a disruption of the intercellular junctions, thus enabling the bacteria to access the basolateral domain of the endothelial cells. Indeed, several reports indicate that *P. aeruginosa* interacts preferentially with basolateral rather than apical domains of target cells [15]. We reasoned that this “priming” by the T2SS should not be effective if cells are sparse at the beginning of infection. Indeed, as shown in Fig. 1E, *P. aeruginosa* action was much faster in these conditions than on confluent cells and there were no significant differences between the cell retraction extents induced by the wild-type or the T2SS-inactive strains at all times p.i. for CHA and PAO1. These features are consistent with a facilitation role of the T2SS, hence promoting the action of the T3SS.

### Endothelial cell-cell junction disruption by *P. aeruginosa*'s secretomes

**Figure 2:**
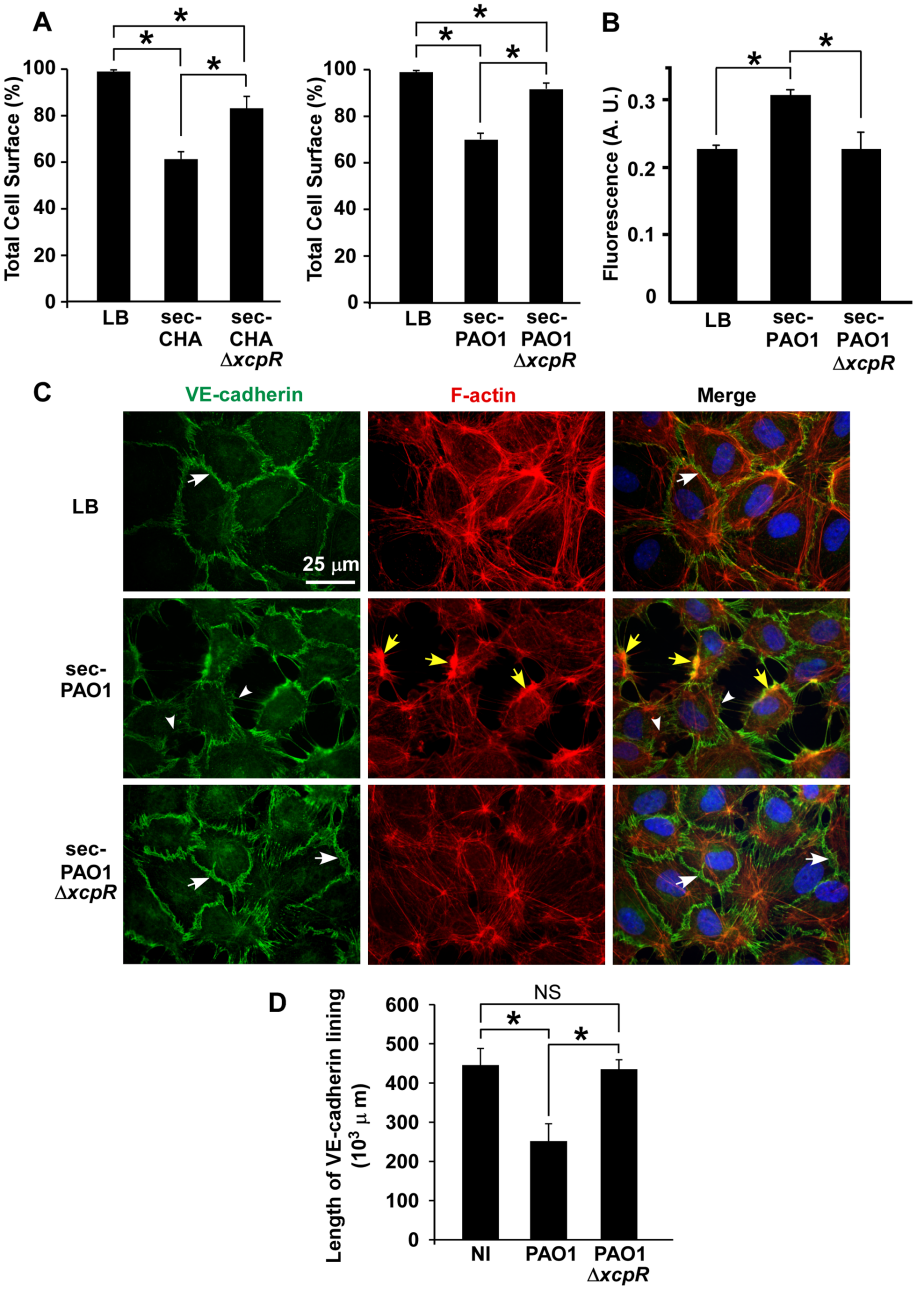
*P. aeruginosa* type 2 effectors provoke gap formation in endothelial cell monolayer. (A) Retraction assay with cells incubated for 1.5 h with 10% volume of secretomes (sec) of CHA (left) and PAO1 (right) strains, in the culture medium. Cell surface was calculated as in Fig. 1A and data represent the mean percentage of cell surface in the field (n = 10)+SD. Statistics: Kruskal-Wallis' test, p<0.001 for each data set; Bonferroni's pairwise post-hoc test showed significant differences in all conditions (*). (B) Monolayer permeability assay with secretomes (10% v/v). LB was used as control. Data are presented as mean fluorescent units (n = 3)+SD. Statistics: 1-way ANOVA, p<0.001; Dunnett's post-hoc test showed significant differences when compared to sec-PAO1 (*). (C) Confluent HUVECs were treated with 10% volume of PAO1 or PAO1Δ*xcpR* secretomes (sec) or LB in the cell culture medium. Cells were fixed 2 h later and labeled for VE-cadherin (green) and F-actin (red). In the merged images, nuclei are also shown in blue. The actin cytoskeleton is present in all 3 conditions. There is however fewer stress fibers and the onset of condensed cortical actin foci (yellow arrows) in retracted cells (sec-PAO1). VE-cadherin antibody decorates cell-cell junctions in LB or in sec-PAO1Δ*xcpR* conditions (arrows), while labeling is attenuated or absent in presence of sec-PAO1 (arrowheads). This alteration parallels gap formation between cells. A similar experiment was performed with CHA secretomes with identical results (Fig. S2). (D) Length of VE-cadherin lining at cell edges was quantified as described in Materials and Methods. Data represent the mean (n = 10)+SD of VE-cadherin lining length. Statistics: 1-way-ANOVA, p<0.001; Bonferroni's pairwise post-hoc test showed significant differences when compared to PAO1 (*), but not between NI and PAO1Δ*xcpR* (NS).

To further characterize the role of T2SS effectors on HUVECs, we incubated cells with PAO1 or CHA secretomes collected in the early stationary growth phase (A_600_ = 1.5) in order to obtain high T2 secretion and low cell lysis. Secretomes tested by zymography yielded expected results in wild-type strains: bands of the main T2S effectors, LasA and LasB, and of AprA, a T1S protease (Fig. S1B) (see [16] for similar zymograms). As anticipated, LasA and LasB activities were minimal in Δ*xcpR* strains (the very faint bands observed for LasA/B in Δ*xcpR* mutants are probably caused by enzyme release from lytic cells). When HUVECs were exposed for 1.5 h to secretomes from wild-type strains we observed a significant cell retraction compared to untreated cells, while this phenomenon was minimal in the presence of secretomes from Δ*xcpR* strains (Fig. 2A). This result was confirmed by data from a permeabilty assay using albumin-TRITC (Fig. 2B). Thus, T2SS effectors are capable of affecting cell morphology independently of the T3SS.

We then addressed the action of secretomes on two important cell morphology components: cell-cell junctions and the actin cytoskeleton. Cells were observed post-incubation with secretomes, after labeling for VE-cadherin, an adhesive protein located at endothelial adherens junctions, and for filamentous (F)-actin (Fig. 2C for PAO1 and Fig. S2A for CHA). Contrary to the known ExoS/ExoT action, the actin cytoskeleton was not disrupted by PAO1 secretomes. However, there were fewer actin fibers and the onset of condensed actin foci at cell cortex, which is consistent with cell retraction. Secretomes from PAO1 or CHA induced gap formation between cells together with loss or attenuation of VE-cadherin labeling at cell edges. In contrast, cell-cell junctions were maintained when these cells were incubated with secretomes from Δ*xcpR* strains. The length of VE-cadherin lining along cell edges was measured and it was indeed lower in the wild-types compared to the T2SS-inactive strains (Fig. 2D and Fig. S2B), further demonstrating that the T2SS effectors decreased VE-cadherin localization at cell edges. Interestingly, *P. aeruginosa* secretomes similarly interfered with ZO-1 labeling, a component of tight junctions (Fig. S3), suggesting that both adherens (VE-cadherin) and tight (ZO-1) junctions are disrupted by T2SS effectors.

### LasB protease is required for VE-cadherin degradation

**Figure 3:**
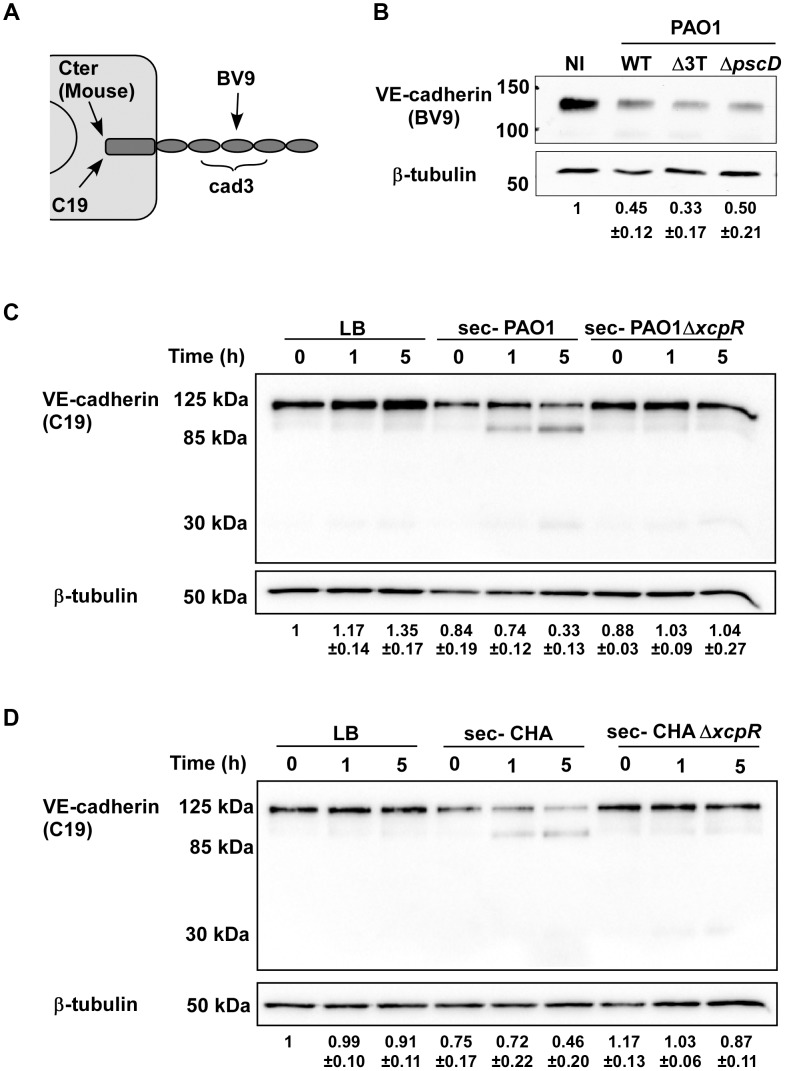
Induction of VE-cadherin proteolytic cleavage by *P. aeruginosa* T2SS. (A) Diagram showing the epitopes recognised by the various VE-cadherin antibodies used in this study. (B) Confluent HUVECs were infected for 4.5 h with PAO1 (WT), PAO1Δ*3Tox* (Δ3T), or PAO1Δ*pscD* (Δ*pscD*) at MOI = 10. Control was non-infected (NI) cells. Protein extracts were analyzed by Western blotting with VE-cadherin (BV9) and ß-tubulin antibodies. Data show a decrease in VE-cadherin levels, occurring in infected cells even in absence of the T3 effectors. (C, D) HUVECs were incubated with secretomes from PAO1, PAO1Δ*xcpR*, CHA, CHAΔ*xcpR* or LB. Protein extracts were prepared at different time points as indicated and analyzed by Western blot with anti-VE-cadherin C-terminus (C19) and ß-tubulin (ß-tub) antibodies. The full-length VE-cadherin band (125 kDa) decreased with time when incubated with wild-type strain secretomes. This was correlated with the appearance of a band at 85 kDa, as well as a faint band at 30 kDa. VE-cadherin cleavage did not occur with Δ*xcpR* strain secretomes or LB. (B–D) The averaged signal ratios (n = 3) ± SD of VE-cadherin (125 kDa) over ß-tubulin are indicated below each lane.

As VE-cadherin is a major determinant of cell-cell junction integrity [17], we examined thereafter the action of bacterial infection on VE-cadherin using various antibodies (Fig. 3A). We first examined VE-cadherin levels in infected HUVECs (Fig. 3B). The data showed decreases in amounts of VE-cadherin in cells when they were infected with PAO1, hence indicating that VE-cadherin is a target of *P. aeruginosa*. Analogous experiments with strains lacking the three T3 toxins (Δ*3T*) or the T3 apparatus (Δ*pscD*) similarly resulted in decreased VE-cadherin levels, thus indicating that this phenomena is independent of the T3SS. To determine whether VE-cadherin was degraded by *P. aeruginosa*-released proteases, HUVECs were incubated with secretomes from wild-type and Δ*xcpR* mutants of PAO1 or CHA strains. Incubation of HUVECs with secretomes from wild-type strains showed the emergence of a band at 85 kDa, as detected by the VE-cadherin antibody directed to the C-terminus (intracellular) part of the protein. This paralleled decreases in the amount of full length protein at 125 kDa (Fig. 3C,D). The 85-kDa band was absent when cells were treated with secretomes from Δ*xcpR* strains or with LB. These results strongly suggest that VE-cadherin is degraded by one or more T2 proteases. Interestingly, intensity of a second faint band at 30 kDa slightly increased with time (Fig. 3C,D). This band may represent a secondary or subsequent cleavage product of *P. aeruginosa* proteases.

**Figure 4:**
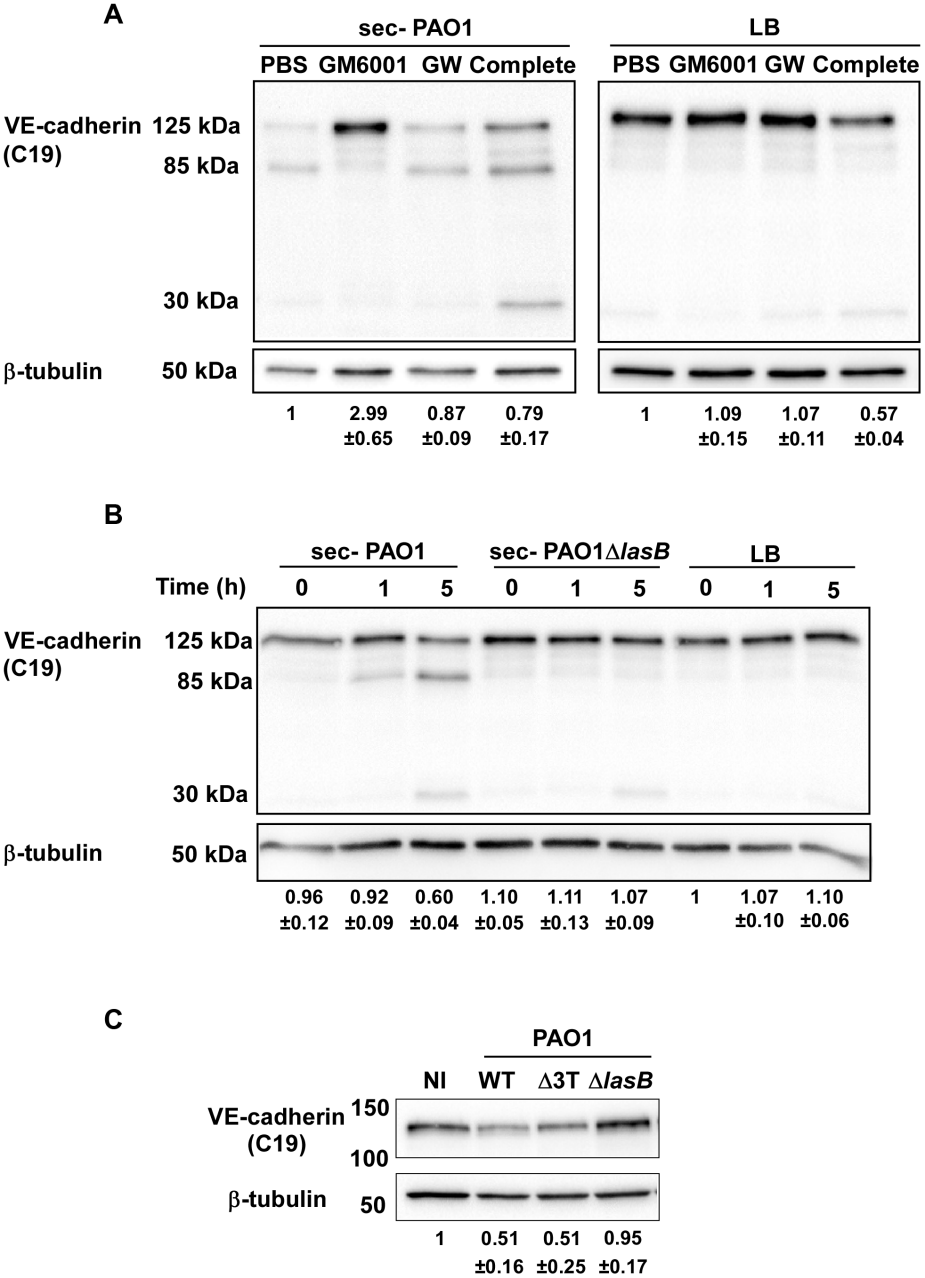
LasB protease is required for VE-cadherin cleavage. (A) PAO1 secretome action on VE-cadherin was tested at 5 hours post-treatment in presence of protease inhibitors: GM6001 for metalloproteases, GW280264X (GW) for ADAMs and the Complete cocktail for serine- and cysteine-proteases. LB was used as control. Conditions are as in Fig. 3. Western blots show that GM6001 was solely capable to prevent VE-cadherin degradation. (B) HUVECs were treated for indicated times with secretomes from PAO1 and PAO1Δ*lasB* strains. Western blot analysis shows that the PAO1Δ*lasB* secretome did not proteolyse VE-cadherin unlike the PAO1 secretome. (C) The direct action of bacterial strains on VE-cadherin was tested at 4 h.p.i. by Western blot as above. The PAO1Δ*lasB* strain induced no degradation compared to the wild type or the Δ*3Tox* strains. (A–C) The averaged signal ratios (n = 3) ± SD of VE-cadherin (125 kDa) over ß-tubulin are indicated below each lane.

To identify the protease(s) involved in VE-cadherin processing, an analogous experiment was performed in the presence of protease inhibitors targeting either (i) metalloproteases (GM6001), (ii) ADAM proteases expressed by endothelial cells that were previously shown to cleave VE-cadherin [18] (GW280264X) or (iii) serine- and cysteine-proteases (Complete cocktail from Roche). The only inhibitor that prevented VE-cadherin cleavage was GM6001 (Fig. 4A), indicating that metalloproteases are solely involved in VE-cadherin processing, and suggesting that *P. aeruginosa* does not operate through activation of endothelial ADAMs.

LasB is a major metalloprotease secreted by *P. aeruginosa*'s T2SS and thus represents a good candidate for induction of VE-cadherin proteolysis [10]. We thus tested a secretome from a PAO1 mutant lacking LasB in a VE-cadherin degradation assay. This secretome lacked both LasB and LasA activity (Fig. S1B), thus confirming that LasB is necessary to activate LasA [19]. The secretome from the PAO1Δ*lasB* strain was unable to degrade VE-cadherin and produce the 85-kDa band (Fig. 4B). We also tested the direct action of the PAO1Δ*lasB* strain on cellular VE-cadherin (Fig. 4C). The VE-cadherin signal after PAO1Δ*lasB* infection was higher than after PAO1 or PAO1Δ3Tox infection and comparable to the uninfected condition. Taken together, we conclude that LasB and/or LasA, are required for VE-cadherin degradation.

### LasB specifically cleaves the VE-cadherin extracellular domain

**Figure 5:**
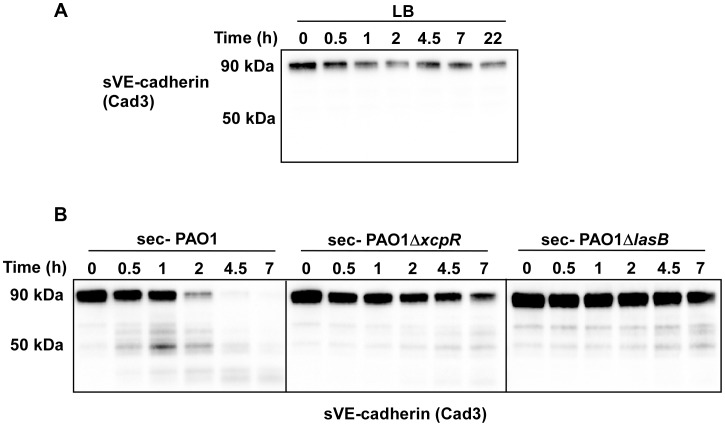
*P. aeruginosa*'s secretomes cleave VE-cadherin extracellular domain. Recombinant soluble VE-cadherin (sVE-cadherin) was incubated at 37°C in presence of LB (A), PAO1, PAO1Δ*xcpR* or PAO1Δ*lasB* (B) secretomes. VE-cadherin was analyzed by Western blot at different time points using Cad3 antibody directed against VE-cadherin extracellular domain. Data show a LasB-dependent VE-cadherin proteolysis.

To confirm that VE-cadherin is a substrate of *P. aeruginosa* secretomes, we produced and purified the glycosylated extracellular domain of VE-cadherin in eukaryotic cells (Fig. S4). This domain was indeed cleaved by wild-type *P. aeruginosa*'s secretomes in a time-dependent manner (Fig. 5). In contrast, only minimal degradation was observed with PAO1Δ*xcpR* and PAO1Δ*lasB* secretomes at a late incubation time (7 h post treatment). Moreover, purified VE-cadherin was unaffected by overnight incubation with LB. The size of the major degraded band (50 kDa) of the VE-cadherin extracellular domain (90 kDa) is consistent with the degraded band size (85 kDa) of the full length protein (125 kDa). In conclusion, T2 effectors, and more specifically LasB, have a direct proteolytic effect on VE-cadherin that is independent of endothelial cell machinery.

**Figure 6:**
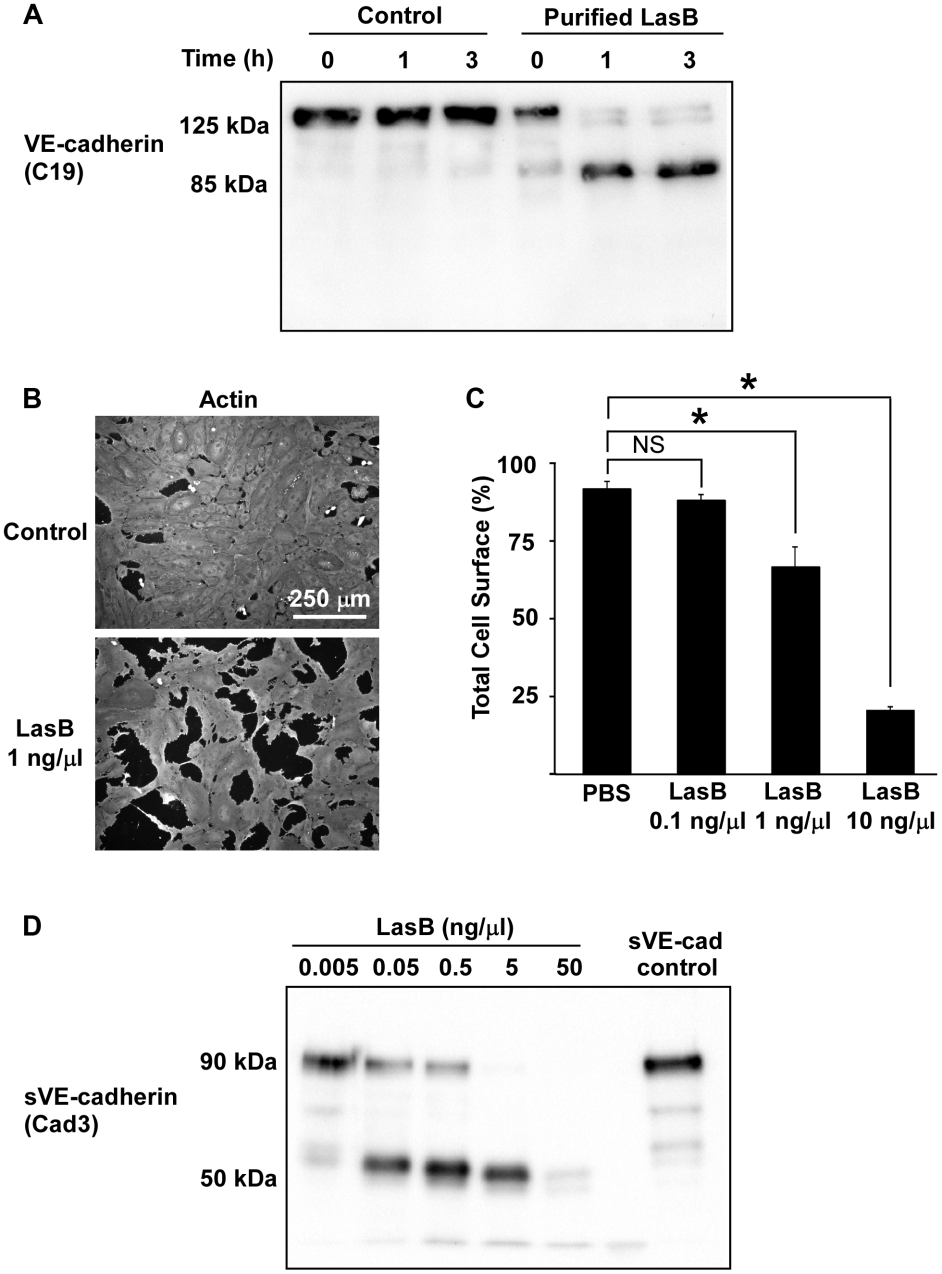
LasB cleaves cellular and soluble VE-cadherin. (A) LasB was added to HUVEC media at 1 ng/µL; protein extracts were prepared at indicated times and analyzed by Western blot with VE-cadherin antibody C19. LasB yielded a cleavage product of similar size (85 kDa) as secretomes. (B) Actin immunofluorescent images of confluent HUVECs treated with LasB (1 ng/µL in culture medium) or untreated (control). LasB induced cell retraction. (C) Quantification of cell spreading using increasing concentrations of LasB. Statistics: 1-way ANOVA: p<0.001; Dunnett's post-hoc test showed significant differences with PBS (*). (D) Purified VE-cadherin extracellular domain (sVE-cadherin) was incubated with various concentrations of LasB and analyzed by Western blot using VE-cadherin antibody Cad3. VE-cadherin cleavage product by LasB is of identical size (50 kDa) as secretomes.

We then aimed to determine whether VE-cadherin was directly cleaved by in vitro-purified LasB or by another protease that could be activated by LasB. We thus incubated HUVECs with purified LasB (Fig. S5) and observed a time-dependent increase of the 85 kDa band (Fig. 6A). Like secretomes, purified LasB also promoted gap formation between cells (Fig. 6B,C). Importantly, LasB cleaved purified VE-cadherin and yielded a degraded band at 50 kDa (Fig. 6D). Altogether, our data show that LasB is the protease responsible for direct VE-cadherin cleavage and suggest that this protease causes cell-cell junction disruption in HUVECs.

**Figure 7:**
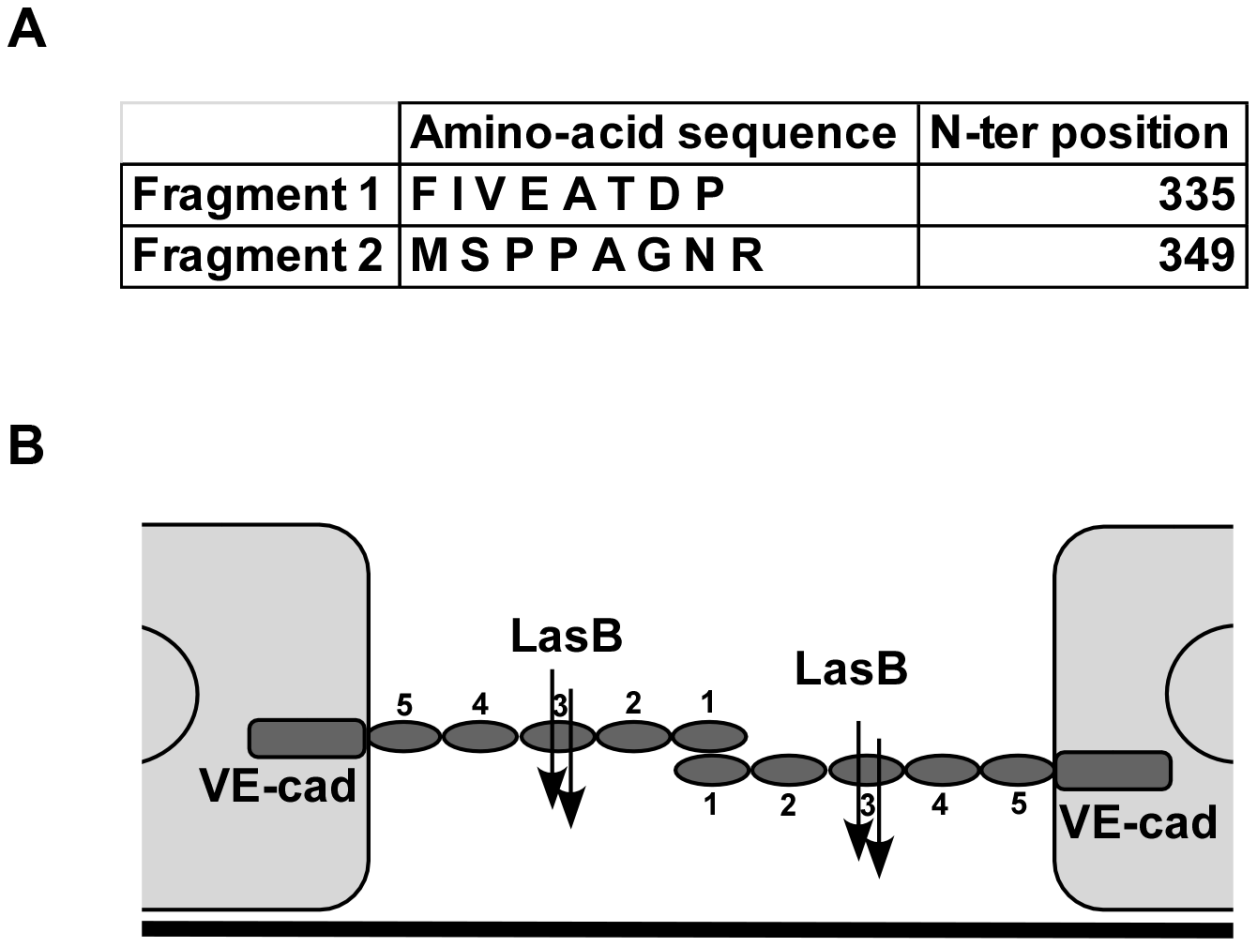
LasB cleavage sites in VE-cadherin extracellular domain. (A) Purified VE-cadherin extracellular domain was incubated with LasB as in Fig. 6 and the N-terminal sequences of protein present in the 50 kDa band were determined. Two sequences were obtained as indicated, both belonging to VE-cadherin sequence. N-terminal positions in VE-cadherin sequence are indicated. (B) Representation of LasB cleavage sites on 2 VE-cadherin molecules located on 2 adjacent endothelial cells. VE-cadherin extracellular domain is constituted by 5 cadherin domains numbered 1-5 from the N-terminus. VE-cadherin homotypic adhesion is promoted by domain 1. LasB cleaves at 2 close positions in the middle of domain 3, precluding endothelial homophilic adhesion.

To further characterize the LasB proteolytic mechanism acting on VE-cadherin, N-terminal sequencing was performed on the 50-kDa fragment. Data showed that LasB cleaves VE-cadherin at two close positions, before amino-acids 335 and 349, in the middle of extracellular cadherin domain 3 (Fig. 7). Therefore, the cleaved protein still linked to endothelial cells by its transmembrane domain lacks the adhesive domain located in cadherin domain 1.

**Figure 8:**
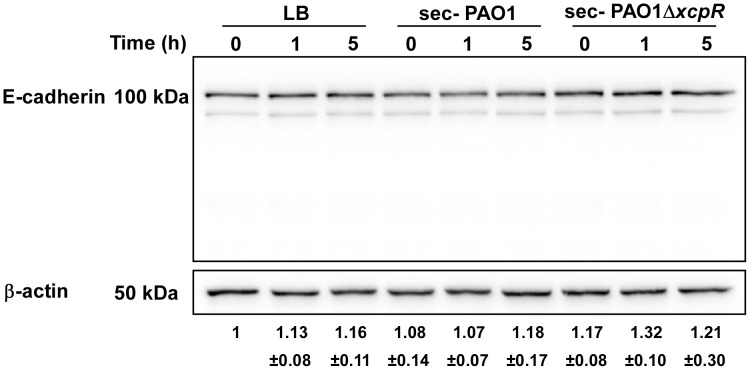
E-cadherin is resistant to LasB proteolysis. Epithelial A549 cells were treated with PAO1 or PAO1Δ*xcpR* secretomes (10% v/v in culture medium). LB was used as control. Proteins were extracted at different time points p.i., as indicated, and analyzed by Western blot with anti-E-cadherin and anti-ß-actin antibodies. E-cadherin was stable and no degradation products were observed. The averaged signal ratios (n = 3) ± SD of E-cadherin (125 kDa) over ß-tubulin are indicated below each lane.

VE-cadherin is specific to endothelial cells and we wondered if T2SS proteases would act similarly on epithelial (E)-cadherin to disrupt epithelial barriers. Contrary to its effect on VE-cadherin, *P. aeruginosa*'s secretome did not degrade E-cadherin in epithelial A549 cells (Fig. 8). The effect of *P. aeruginosa*'s T2SS on cell-cell junction disruption is thus specific to endothelial cells.

### VE-cadherin degradation in *P. aeruginosa*-induced pneumonia

To study VE-cadherin stability during in vivo infection, we used a mouse model of acute pneumonia. To eliminate potential interference with neutrophil-released proteases known to cleave VE-cadherin [20], neutropenia was induced by injection of cyclophosphamide (100 mg/Kg IP) for 4 days prior to infection. Effectiveness of neutropenic treatment was verified on blood smears and on histological sections of infected lungs (data not shown).

**Figure 9:**
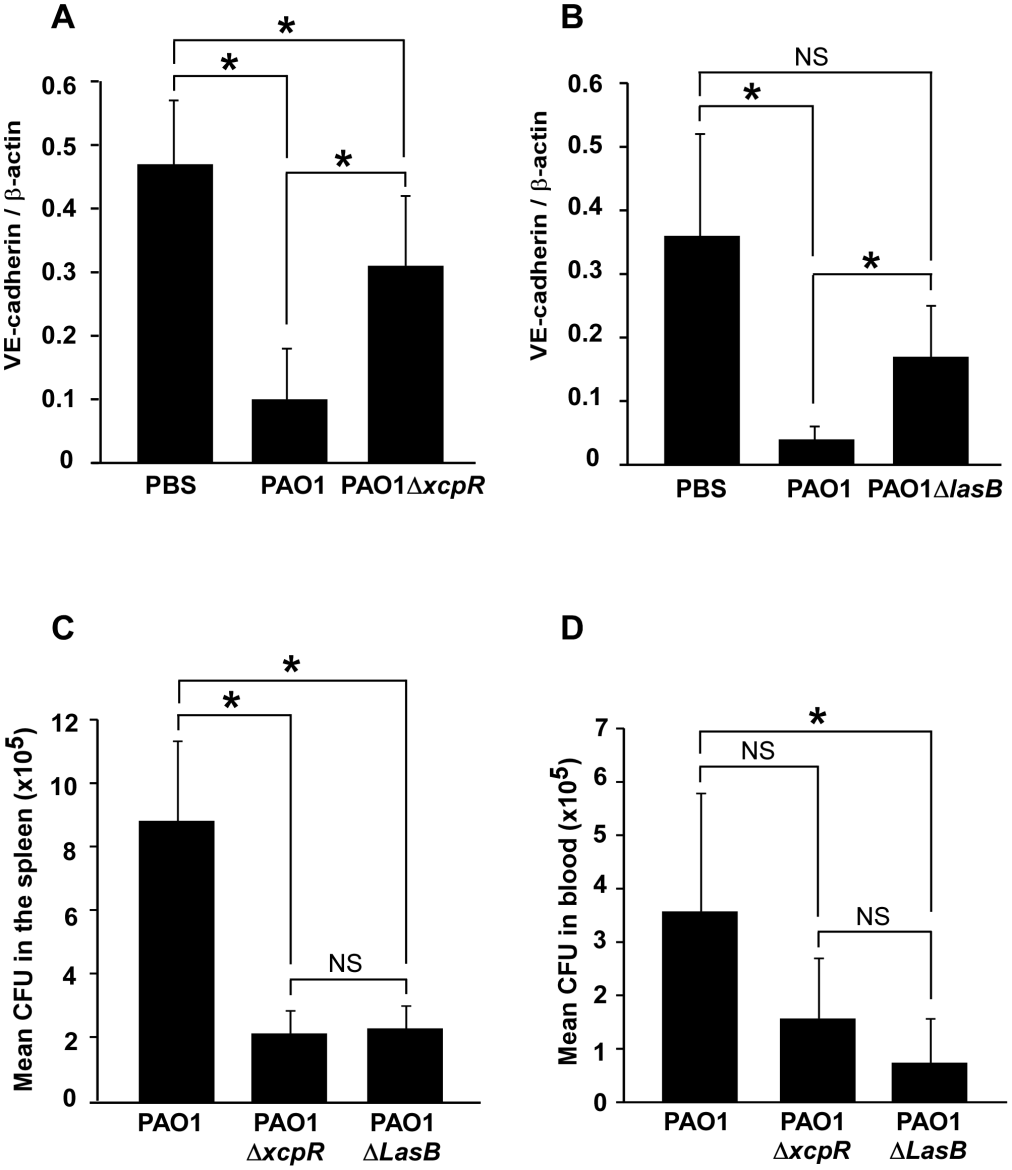
Decreased VE-cadherin levels in mouse acute pneumonia correlated with increased dissemination. (A) Mice were infected by airway instillation of a suspension (2.5. 10^6^) of PAO1, PAO1Δ*xcpR* or mock-infected with PBS. (B) Similar experiment with PAO1Δ*LasB* mutant strain. Lungs were resected 16 hours later and proteins were analyzed by Western blot using antibodies against mouse VE-cadherin C-terminus (VE-cad Cter) and ß-actin (ß-act). Signals were quantified and data are presented as mean (n = 5) VE-cadherin/ß-actin signal ratios+SD. VE-cadherin levels were strikingly diminished in PAO1-infected lungs, while those infected with PAO1Δ*xcpR* or PAO1Δ*LasB* only displayed a moderate decrease. Statistics for (A): 1-way ANOVA, p<0.001, for (B): Kruskal-Wallis, p = 0.004. Pairwise significance (*) was determined by Bonferroni's test. (C, D) Mice were infected by airway instillation of a suspension of PAO1, PAO1Δ*xcpR* or PAO1Δ*LasB* (2.5. 10^6^). Blood samples and spleen were withdrawn 16 hours later. *P. aeruginosa* colony forming units (CFU) were evaluated in both locations by conventional techniques. Data are presented as the mean CFU (n = 5)+SEM, calculated for total spleens (C) or total mouse blood (D). Statistics for (C): Kruskal-Wallis, p = 0.008, for (D): 1-way ANOVA, p = 0.032. Pairwise significance (*) was determined by Bonferroni's test. Data are representative of 3 independent experiments.

A suspension of either PAO1, PAO1Δ*xcpR* or PAO1Δ*lasB* was instilled in mouse airways, and lungs were resected 16 h later. VE-cadherin levels were strikingly lower in wild-type-infected lungs compared to mock-infected lungs (Fig. 9A,B). Moreover, infection with PAO1Δ*xcpR* or PAO1Δ*lasB* induced a significantly smaller decrease in VE-cadherin levels, indicating that the *P. aeruginosa*'s T2SS, and more specifically LasB, promoted VE-cadherin degradation in vivo. This result was similar to what was observed in HUVECs. However, no degradation band was detected (not shown), suggesting that proteolytic mechanisms might be different in mice and humans. Alternatively it is possible that in vivo cleavage by T2SS effectors is rapidly followed by other proteolytic events acting on T2SS-induced degradation products.

We then reasoned that if *P. aeruginosa*'s T2SS promoted VE-cadherin cleavage in such a dramatic manner, it should probably induce endothelial cell-cell junction disruption and thus have an impact on bacterial dissemination throughout the body. Therefore, we compared *P. aeruginosa* colony forming units (CFU) in blood and spleen 16 hours post-infection (Fig. 9C,D). In both locations, we found less CFU when mice were infected with PAO1Δ*xcpR* or PAO1Δ*lasB* compared to the wild-type strain, although this difference was not statistically significant in the blood for PAO1Δ*xcpR*.

In conclusion, *P. aeruginosa's* T2SS influenced bacterial dissemination in the mouse via a mechanism that may involve VE-cadherin degradation by T2SS proteases, thereby affecting endothelium barrier properties.

## Discussion

Rupture of the endothelial barrier by *P. aeruginosa* requires the joint action of several virulence factors [8]. Here we show that a T2SS effector, the protease LasB, might be one of the primary weapons in this arsenal required for disruption of endothelium integrity.

We found that T2SS-competent bacteria induced faster cell retraction than bacteria lacking a T2SS. Dislocation of the actin cytoskeleton and subsequent cell retraction are known to be T3SS-mediated. We thus wondered how secreted toxins (T2 effectors) could cooperate with internal exotoxins (T3 effectors) to promote cytoskeleton collapse. As *P. aeruginosa*'s secretomes could induce gap formation between cells, but not cytoskeleton disruption, we hypothesized that secreted toxins could directly act on adhesive proteins located at cell-cell junctions. Indeed, we showed that LasB protease cleaves VE-cadherin, a major adhesive protein of interendothelial junctions. This was demonstrated by the use of a LasB deletion mutant and confirmed by purified LasB. Importantly, most of these experiments were performed with two strains of different origins: one (PAO1, ATCC 15692) isolated from a wound and the other (CHA) from the lungs of a cystic fibrosis-patient [21]. Their actions on VE-cadherin were comparable, thereby suggesting that this is a common feature among *P. aeruginosa* subtypes.

VE-cadherin is required to maintain cell interaction in the mature endothelium, as demonstrated by numerous *in vitro* and *in vivo* experiments, including low-calcium switch (VE-cadherin adhesive activity is calcium-dependent) and the addition/administration of VE-cadherin adhesion-blocking antibodies to cells or mice [22]–[24]. The rupture of cell-cell junctions by LasB-induced VE-cadherin cleavage is consistent with these data.

Interestingly, VE-cadherin cleavage disrupted endothelial adherens junctions but also induced little but significant cell retraction with minor modifications to the actin cytoskeleton architecture (Fig. 1A,B and 2C). The T2SS-dependent disruption of monolayer integrity was confirmed by analysis of albumin-TRITC diffusion and bacterial transmigration across the monolayer (Fig. 1C,D and 2B). The intercellular junctions are subjected to two opposite forces: one centrifugal mediated by homophilic adhesive proteins and the other centripetal resulting from acto-myosin contraction [25]. Thus, rupture of intercellular junctions may induce acto-myosin-dependent retraction without any further action from the bacteria.

Our findings with the acute pneumonia model indicate that mouse VE-cadherin is also proteolysed in infected lungs by a LasB-dependent mechanism (Fig. 9A,B). Bacteria lacking a functional T2SS or LasB are less prone to dissemination (Fig. 9C,D); it is therefore likely that LasB-dependent VE-cadherin cleavage participates in bacteria dissemination. Mechanisms of mouse VE-cadherin degradation deserve further investigation. In particular, it will be of interest to know in which vessel type LasB acts on VE-cadherin and how this phenomenon correlates with increased vascular permeability.

As far as we know, this is the first time that a *P. aeruginosa* T2SS mutant has been assayed for dissemination in a mouse pneumonia model. However, it was previously reported that co-injection of LasB with a non-lethal inocula of strain PA103 in mouse muscles induced mortality and increased dissemination in the liver [26], suggesting that LasB contributes to the invasiveness of the organism. Conversely, LasB did not play any role in a burn infection mouse model [27].

The tight junction protein ZO-1 also vanished from cell-cell junctions in a T2SS-dependent manner. Tight junctions are known to be dismantled once VE-cadherin-dependent adherens junctions are cleared [28]. It is thus likely that tight junction disruption is subsequent to VE-cadherin proteolysis. However, one cannot exclude that tight junction adhesive proteins be also cleaved or downregulated by T2 effectors. Indeed, LasB was shown to increase monolayer permeability and to induce depletion of ZO-1 and ZO-2, both required for tight junction assembly, in MDCK epithelial cells [29].

VE-cadherin loss by LasB-mediated cleavage may be exacerbated by the release by the T2SS of exotoxin A, an ADP-ribosyl transferase that inhibits protein synthesis by targeting elongation factor-2 [10]. Hence, exotoxin A may prevent VE-cadherin resynthesis and thus preclude reconstruction of the junctions.

Integrity of the main barriers in mammalian bodies is dependent upon E-cadherin in epithelia and VE-cadherin in endothelia. Here we show that LasB-dependent cadherin cleavage is specific to VE-cadherin; E-cadherin was resistant to LasB, thus indicating that *P. aeruginosa*-induced epithelial barrier breakdown does not proceed by an analogous mechanism. Recently, it has been reported that a quorum sensing molecule, 30-C_12_-HSL, can disrupt epithelial barrier integrity, in addition to its role in virulence factor regulation in bacteria [30]. 30-C_12_-HSL increased phosphorylation of major components of tight and adherens junctions, a process known to augment paracellular permeability [31]. Bacterial secretion of this molecule may thus provide a supplementary mechanism for junction opening.

LasB is known to proteolyse several extracellular proteins including elastin, fibronectin, vitronectin, surfactant proteins and serum complement components [10], [11], [32]–[36]. Extracellular matrix protein degradation by LasB was shown to induce anoikis of aortic valve myofibroblasts [36]. In contrast, LasB was shown to cleave a limited number of surface receptors: proteinase activated receptor-2 in respiratory epithelial cells and urokinase plasminogen activator receptor in monocytic and respiratory epithelial cells [37], [38]. Conversely, integrins are resistant to LasB proteolysis [36]. Altogether, these data indicate that LasB is not a multisubstrate proteolytic enzyme for cellular proteins, but instead targets specific receptors. Limitation of action of bacterial virulence factors as opposed to killing bacteria is now recognized as a promising antimicrobial strategy. Inhibition of LasB activity using a peptide based on the LasB cleavage site in the VE-cadherin sequence may thus be of clinical interest to abate bacterial propagation.

**Figure 10:**
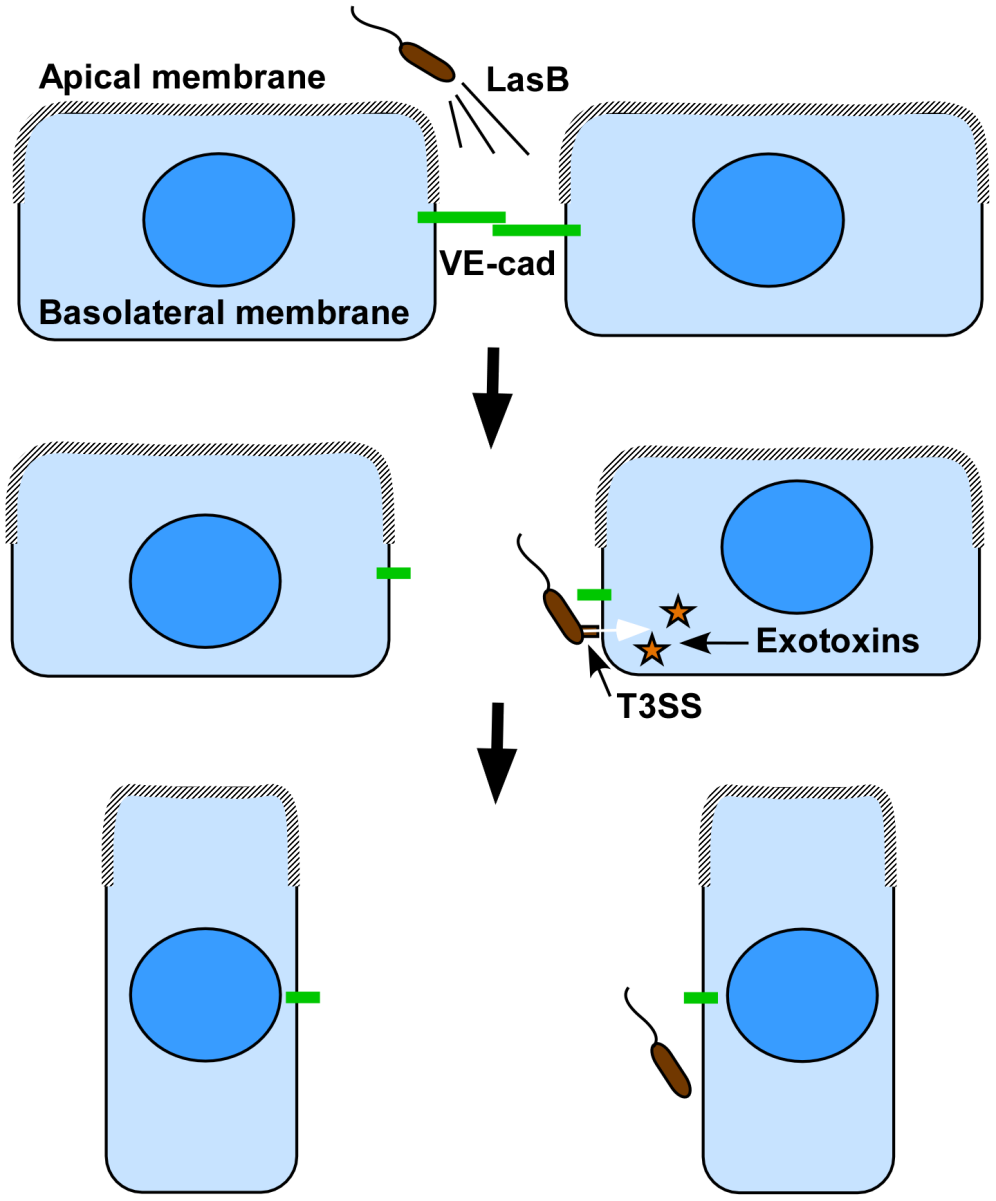
Proposed cooperation model between *P. aeruginosa*'s T2SS and T3SS. As previously reported in epithelial cells, the apical membrane may not be permissive to bacterial interaction with endothelial cells, thus preventing T3SS-dependent intoxication. Our model proposes that bacteria spray T2SS proteases, including LasB, in close vicinity of cell-cell junctions, leading to VE-cadherin cleavage and junction opening. In addition to the induced rupture in endothelium barrier integrity, cell-cell junction disruption gives bacterium access to the basolateral membrane, a more favorable domain for bacterial landing and exotoxin injection. Injection of T3 effectors leads to cell retraction and a complete loss of the endothelial barrier.

We noticed that HUVEC intoxication either by CHA or PAO1 was more delayed in confluent cells than in sparse cells (Fig. 1B,E), indicating that cells in a reconstituted endothelium have more resistance to *P. aeruginosa* infection than sparse cells. The retraction effect of the T2SS and the T3SS were not simply additive, but synergistic (Fig. 1B), suggesting a real cooperation between the two systems. Furthermore, cell retraction was not synchronized in the confluent monolayer, but began and propagated from specific points (Fig. S6). This occurred even with high amounts of bacteria (MOI of 10 or above) and thus with the capacity to infect all cells simultaneously. Recently, Kierbel *et al.* [15] showed that the apical domain of epithelial cells was not a favorable bacteria landing surface compared to the basolateral domain. A similar phenomenon may occur with endothelial cells, as suggested by the unsynchronized infection process. Following this hypothesis, LasB-dependent junction breaches may allow bacteria access to the basolateral domain at specific points. We thus propose a 2-step model of endothelial infection, in which (i) LasB is secreted in the vicinity of cell-cell junctions leading to gap formation and (ii) the bacteria gain access to the cell basolateral domain at interrupted junctions, thus allowing host-pathogen interaction and T3SS-dependent intoxication (Fig. 10). In this context, T2SS-dependent junction opening may be a limiting step of endothelial infection and vascular barrier breakdown. In fact, the roles of *P. aeruginosa*'s T2SS and T3SS are usually studied separately and their secreted virulence factors are often considered to work independently even though these two secretion systems were shown to have a cumulative toxic effect in mouse models of infection [3]. Here, we provide a molecular mechanism that accounts for cooperation between these two types of virulence factors.

In conclusion, we identified a novel substrate for *P. aeruginosa*'s LasB protease and a mechanism of action that may be of major importance for bacterial dissemination and generation of septicemia. As VE-cadherin has a pivotal role in endothelial barrier integrity, its degradation during the infection process probably leads to dramatic endothelial dysfunction, comparable to that reported after injection of VE-cadherin-blocking antibodies. Furthermore, it facilitates T3SS-dependent cell intoxication and thus significant endothelium damage. In this context, T2-dependent junction opening may be a limiting and critical step of endothelial infection and vascular barrier breakdown.

## Materials and Methods

### Ethics statement

All animal protocols in this study were conducted in strict accordance with the French guidelines for the care and use of laboratory animals (Charte nationale portant sur l'éthique de l'expérimentation animale, January 20, 2009). The protocol for mouse infection was approved (project license number 12-051) by the animal research committee of the institute (CETEA).

### Reagents

The Complete protease inhibitor cocktail was from Roche and the metalloprotease inhibitor GM6001 from Santa Cruz. The ADAM inhibitor GW280264X was a generous gift from Pr Andreas Ludwig (Aachen, Germany). Antibodies against human VE-cadherin extracellular domain (Cad3 and BV9) were gifts from Danielle Gulino-Debrac (Grenoble, France) and Annunciata Vecchi (Milan, Italy), respectively. Antibody against human VE-cadherin C-terminal domain was purchased from Santa-Cruz (C-19) and that against the mouse C-terminal domain was produced against the peptide CSDPQEELI. ß-tubulin and ß-actin antibodies were from Sigma and E-cadherin antibodies from Life Technologies. Phalloidin-TRITC was from Sigma-Aldrich. Peroxidase-conjugated antibodies were purchased from Biorad laboratories. The secondary antibodies coupled with Alexa 488 were purchased from Invitrogen. Purified *P. aeruginosa* LasB was purchased from Nagase ChemteX Corp. (Kyoto, Japan) and purity was evaluated by electrophoresis and Coomassie staining (Fig. S5).

### Bacterial culture and strains

The non-polar chromosomal gene deletion of *xcpR* in *P. aeruginosa* strains PAO1 and CHA was obtained by double cross over events and pKNG101 suicide vector properties as previously described [39]. Briefly, 500-bp upstream and downstream regions of *xcpR* gene were PCR amplified, and tandemly cloned into pKNG101vector. The resulting construct, pKN-*ΔR* was transferred by conjugation into wild type *P. aeruginosa* PAO1 and CHA strains in order to generate the *xcpR* mutants. The strain in which the chromosomal integration event occurred was selected on *Pseudomonas* isolation agar plates containing 2 mg of streptomycin per mL. Excision of the plasmid, resulting in the deletion of the chromosomal target gene, was performed after selection on LB plates containing 5% sucrose. Colonies that became sucrose resistant and streptomycin sensitive were confirmed to contain the gene deletion by PCR analysis. Deletion of *lasB* in PAO1 and *pscF* in CHA were described previously (see Table 1). PAO1F, PAO1Δ*3Tox* and PAO1Δ*pscD* were generous gifts from Arne Rietsch. *P. aeruginosa* was grown in liquid Luria Broth (LB) medium at 37°C with agitation or on *Pseudomonas* Isolation Agar plates (PIA; Difco). Incubation was performed at 37°C with agitation until the cultures reached *A_600_* values of 1.0 for bacterial infection or 1.5 for secretome production. Secretomes were aliquoted and stored at −80°C after centrifugation and filtration on 0.22 µm filters.

### Zymography

Zymography was performed using standard procedures. Briefly, protein electrophoresis was performed in non-reducing SDS-PAGE containing 0.2% gelatin (calf skin type IV from Sigma-Aldrich). Gel was first incubated in 2.5% Triton-X100 for 1 h and then in 0.2 M Tris pH 7.8, 5 mM CaCl_2_ for 18 h at 37°C. Presence of protein was revealed by Coomassie staining.

### Cell culture

Human umbilical vein endothelial cells (HUVECs) were isolated according to previously described protocols [8]. HUVECs were cultured in Endothelial-Basal-Medium (EBM-2, Lonza) supplemented as recommended by the manufacturer. The epithelial lung carcinoma cell line A549 was grown in RPMI medium with 10% fetal calf serum. Serum was omitted in all infection experiments. In all experiments, cells were cultured for 3 days at confluency; conditions which are sufficient to induce mature adherens and tight junctions (hallmarks of polarized cells), i.e. continuous junctional labeling of VE-cadherin/E-cadherin and ZO-1, respectively.

### Immunofluorescence staining and cell retraction assay

For VE-cadherin and F-actin stainings, cells were fixed with 4% paraformaldehyde for 15 min and permeabilized by 0.5% triton X-100 for 5 min. Cells were then stained using standard procedure with appropriate primary and secondary antibodies or phalloidin-TRITC. Stained cells were observed with a Zeiss epifluorescence microscope (Axioplan).

For the cell retraction assay, cells were fixed in methanol at −20°C for 10 min and labeled for actin and with Hoechst for cell count. Images were captured with low magnification objective (16×) and treated with ImageJ software [8]. Briefly, images of actin staining were binarized and the total cell surface was calculated for each image (n = 10). For experiments with sparse cells, the total cell surface was divided by the cell number in the field. The total length of VE-cadherin lining along cell edges was measured with ImageJ software. Briefly, images of VE-cadherin labeling were binarized and skeletonized. The total length of the skeleton was then measured.

### Western blots

Cells and tissues were lysed in Triton X-100 and protein concentration of the lysates was determined with a Micro-BCA kit (Pierce, Rockford, IL) using BSA as standard. Protein extracts were then separated by SDS-PAGE, transferred onto Hybond ECL membrane (Amersham Biosciences, Saclay, France) and revealed with the appropriate antibody using standard procedures. Signals were revealed with a ChemiDoc system (BioRad). Band intensities were quantified on non-saturated images using imageJ software.

### Transendothelial albumin flux and bacterial transmigration

For albumin flux, measurement was done using established procedures. Briefly, HUVECs were seeded onto tissue culture inserts containing porous membranes (Greiner Thincert 0.4 µm). Once confluent, cells were infected as described above by addition of bacteria or secretomes in the upper compartment. Five micrograms of bovine serum albumin coupled to TRITC (Nordic Immunological) were added together with bacteria or secretomes in the upper compartment. Fluorescence in the lower compartment was measured at 7 hours p.i. in a fluorescence plate reader (ThermoScientific). In case of bacterial infection, bacteria transmigration was examined by plating of serial dilutions of the lower compartment medium on PIA plates and CFU counting. Results shown were calculated for the total volume of the lower compartment.

### Production of VE-cadherin extracellular domain

HEK-293-EBNA cells producing soluble VE-cadherin were kindly provided by Danielle Gulino-Debrac (Grenoble, France). The recombinant protein consists of human VE-cadherin extracellular domain (amino-acids 1-593) fused at its C-terminus to 6 histidines for chromatography purification on Ni-column. Briefly, the cell conditioned medium was harvested, dialyzed against Tris 50 mM pH 8.0 and subjected to ion exchange chromatography on HiTrap Q HP (Akta purifier). The elution fractions were further purified by chromatography on Ni-columns. The eluted protein was evaluated for purity by coomassie staining (Fig. S4) and Western blotting (not shown).

### N-terminal sequencing of VE-cadherin cleavage products

Recombinant VE-cadherin was exposed to purified LasB and reaction products were electrophoresed, transferred on PVDF membrane and stained with Coomassie. The 50 kDa band was excised and subjected to N-terminal sequencing. Amino acid sequence determination based on Edman degradation was performed using an Applied Biosystems gas-phase sequencer model 492. Phenylthiohydantoinamino acid derivatives generated at each sequence cycle were identified and quantitated on-line with an Applied Biosystems Model 140C HPLC system using the data analysis system for protein sequencing from Applied Biosystems Model 610A. Chromatography was used to identify and quantify the derivatized amino acid removed at each sequence cycle. Retention times and integration values of peaks were compared to the chromatographic profile obtained for a standard mixture of derivatized amino acids

### *P. aeruginosa*-induced lung injury

Pathogen-free Balbc male mice (8–10 weeks) were obtained from Harlan Laboratories and housed in the CEA animal care facility. Neutropenia was induced by cyclophosphamide administration (100 mg/Kg IP) for 4 days (days 1–4). At day 5, bacteria in exponentional growth phase were centrifuged and resuspended in sterile PBS at 0.85.10^8^ per mL as evaluated by spectroscopy. Mice were anesthetized by intraperitoneal administration of a mixture of xylazine (10 mg/Kg) and ketamine (50 mg/Kg). Then, 30 µL of bacterial suspension (i.e. 2.5.10^6^ bacteria) were deposited into mouse nostrils. Mice were euthanized by CO_2_ inhalation 16 hours later. Lungs were resected and lyzed for protein extraction. Blood samples and spleen were withdrawn and spleen was homogenized in 2 mL PBS in a Polytron. Serial dilutions of blood and spleen homogenate were spread onto PIA plates for CFU determination.

### Statistics

Multiple comparison tests (either ANOVA or Kruskal-Wallis depending on data distribution) and post-hoc tests (either pairwise or relative to control) were performed as indicated in the legends, using SigmaPlot software.

### Accession numbers of genes and proteins mentioned in this study

Human proteins: VE-cadherin (CAA56306), E-cadherin (CAA84586), ZO-1 (Q07157). *P. aeruginosa* proteins: AprA (AFX88325), LasA (AAC12656), LasB (AEM45937), PscD (AAC44775), PscF (AAC44777), XcpR (AAB27772).

## Supporting Information

Figure S1 **Type 2 and type 3 secretions of** ***P. aeruginosa*** **strains.** (A) Secretomes (at *A_600_* = 1.0) were produced in LB (Non induced) or in LB supplemented with 5 mM EGTA to induce T3 effector secretion (Induced). The secretomes (16 µL) were analyzed by Western blot using ExoS antibodies. ExoS was secreted by all analyzed strains in induced conditions. (B) Secretomes (10 µL) collected at *A_600_* = 1.5 were analyzed by electrophoresis in gelatin-containing SDS-PAGE. After overnight incubation, in-gel protein degradation was assayed by coomassie staining. Protease electrophoretic pattern was as previously reported [16]. LasA and LasB activities were minimal in Δ*xcpR* strains, but also in the LasB-deficient strain because of the required LasB activation of LasA [19]. Remaining LasA/LasB activities observed in Δ*xcpR* strains probably originate from lysed bacteria. Activity of AprA, a T1SS protease, was present in all secretomes.
(PDF)

Figure S2 ***P. aeruginosa*** **'s type 2 effectors provoke gap formation in endothelial cell monolayer.** (A) Confluent HUVECs were treated with a 10% volume of CHA or CHAΔ*xcpR* secretomes (sec) or LB in the cell culture medium. Cells were fixed 1.5 hours later and labeled for VE-cadherin (green) and filamentous (F)-actin (red). In the merged images, nuclei are also shown in blue. The actin cytoskeleton is present in all 3 conditions, however, as for PAO1, there were fewer actin fibers and the onset of condensed cortical actin foci (yellow arrows) in retracted cells (sec-CHA). The VE-cadherin antibody decorates cell-cell junctions in LB or in sec-CHAΔ*xcpR* conditions (arrows), while labeling is attenuated or absent in presence of sec-CHA (arrowheads). This alteration parallels gap formation between cells. A similar experiment was performed with PAO1 secretomes with identical results (Fig. 2). (B) Length of VE-cadherin lining at cell edges was quantified on 10 images for each condition. Data represent the mean+SD of VE-cadherin lining length. Statistics: 1-way ANOVA, p<0.001; Significance was determined using pairwise Bonferroni's test (*).
(PDF)

Figure S3 **Secretomes from** ***P. aeruginosa*** **disrupt tight junctions.** Confluent HUVECs were treated for 3 hours with PAO1 or CHA secretomes either wild type or Δ*xcpR* as indicated. Controls were noninfected cells (NI). Cells were fixed and labeled for F-actin (red) or ZO-1 (green), an essential tight junction component. ZO-1 antibody produced a very thin staining at cell-cell junctions (arrows); in presence of secretomes from wild-type strains, labeling disappeared (arrowheads).
(PDF)

Figure S4 **Electrophoretic analysis of purified soluble VE-cadherin.** VE-cadherin extracellular domain fused to 6-histidine tag (sVE-cad) was produced in HEK-293 cells. Recombinant protein was purified from conditioned medium by ion exchange and nickel-histidine affinity chromatographies successively. Purified protein was electrophoresed and gel was Coomassie stained. Data shows a major band at 90 kDa and a very faint band at 25 kDa.
(PDF)

Figure S5 **Electrophoretic analysis of purified LasB.** Electrophoretic analysis and silver staining of purified LasB shows one band at 35 kDa.
(PDF)

Figure S6 **Propagation of cell retraction in HUVEC monolayer.** Confluent HUVECs were infected for 2 hours with PAO1 at MOI = 10. Cells were fixed and labeled with actin antibody (green). Endothelial cell retraction was not synchronized, but started in specific points and propagated from these sites (arrows).
(PDF)

## References

[1] El Solh AA, Alhajhusain A (2009) Update on the treatment of Pseudomonas aeruginosa pneumonia. The Journal of antimicrobial chemotherapy 64: 229–238. 19520717 10.1093/jac/dkp201

[2] El-Solh AA, Hattemer A, Hauser AR, Alhajhusain A, Vora H (2012) Clinical outcomes of type III Pseudomonas aeruginosa bacteremia. Critical care medicine 40: 1157–1163. 22080633 10.1097/CCM.0b013e3182377906 PMC3288436

[3] Jyot J, Balloy V, Jouvion G, Verma A, Touqui L, et al (2011) Type II secretion system of Pseudomonas aeruginosa: in vivo evidence of a significant role in death due to lung infection. The Journal of infectious diseases 203: 1369–1377. 21502078 10.1093/infdis/jir045 PMC3080898

[4] Vance RE, Rietsch A, Mekalanos JJ (2005) Role of the type III secreted exoenzymes S, T, and Y in systemic spread of Pseudomonas aeruginosa PAO1 in vivo. Infection and immunity 73: 1706–1713. 15731071 10.1128/IAI.73.3.1706-1713.2005 PMC1064930

[5] Deng Q, Barbieri JT (2008) Molecular mechanisms of the cytotoxicity of ADP-ribosylating toxins. Annual review of microbiology 62: 271–288. 10.1146/annurev.micro.62.081307.162848 18785839

[6] Engel J, Balachandran P (2009) Role of Pseudomonas aeruginosa type III effectors in disease. Current opinion in microbiology 12: 61–66. 19168385 10.1016/j.mib.2008.12.007

[7] Hauser AR (2009) The type III secretion system of Pseudomonas aeruginosa: infection by injection. Nature reviews 7: 654–665. 10.1038/nrmicro2199 PMC2766515 19680249

[8] Huber P, Bouillot S, Elsen S, Attrée I (2013) Sequential inactivation of Rho GTPases and Lim kinase by Pseudomonas aeruginosa toxins ExoS and ExoT leads to endothelial monolayer breakdown. Cell Mol Life Sci [epub ahead of print]. 10.1007/s00018-013-1451-9 PMC11113219 23974244

[9] Sayner SL, Frank DW, King J, Chen H, VandeWaa J, et al (2004) Paradoxical cAMP-induced lung endothelial hyperpermeability revealed by Pseudomonas aeruginosa ExoY. Circulation research 95: 196–203. 15192021 10.1161/01.RES.0000134922.25721.d9

[10] Bleves S, Viarre V, Salacha R, Michel GP, Filloux A, et al (2010) Protein secretion systems in Pseudomonas aeruginosa: A wealth of pathogenic weapons. Int J Med Microbiol 300: 534–543. 20947426 10.1016/j.ijmm.2010.08.005

[11] Kipnis E, Sawa T, Wiener-Kronish J (2006) Targeting mechanisms of Pseudomonas aeruginosa pathogenesis. Medecine et maladies infectieuses 36: 78–91. 16427231 10.1016/j.medmal.2005.10.007

[12] Douzi B, Ball G, Cambillau C, Tegoni M, Voulhoux R (2012) Deciphering the Xcp Pseudomonas aeruginosa type II secretion machinery through multiple interactions with substrates. The Journal of biological chemistry 286: 40792–40801. 10.1074/jbc.M111.294843 PMC3220450 21949187

[13] Bardoel BW, Hartsink D, Vughs MM, de Haas CJ, van Strijp JA, et al (2012) Identification of an immunomodulating metalloprotease of Pseudomonas aeruginosa (IMPa). Cellular microbiology 14: 902–913. 22309196 10.1111/j.1462-5822.2012.01765.x

[14] Ball G, Chapon-Herve V, Bleves S, Michel G, Bally M (1999) Assembly of XcpR in the cytoplasmic membrane is required for extracellular protein secretion in Pseudomonas aeruginosa. Journal of bacteriology 181: 382–388. 9882649 10.1128/jb.181.2.382-388.1999 PMC93389

[15] Kierbel A, Gassama-Diagne A, Rocha C, Radoshevich L, Olson J, et al (2007) Pseudomonas aeruginosa exploits a PIP3-dependent pathway to transform apical into basolateral membrane. The Journal of cell biology 177: 21–27. 17403925 10.1083/jcb.200605142 PMC2064102

[16] Tingpej P, Smith L, Rose B, Zhu H, Conibear T, et al (2007) Phenotypic characterization of clonal and nonclonal Pseudomonas aeruginosa strains isolated from lungs of adults with cystic fibrosis. Journal of clinical microbiology 45: 1697–1704. 17392437 10.1128/JCM.02364-06 PMC1933084

[17] Wallez Y, Huber P (2008) Endothelial adherens and tight junctions in vascular homeostasis, inflammation and angiogenesis. Biochimica et biophysica acta 1778: 794–809. 17961505 10.1016/j.bbamem.2007.09.003

[18] Schulz B, Pruessmeyer J, Maretzky T, Ludwig A, Blobel CP, et al (2008) ADAM10 regulates endothelial permeability and T-Cell transmigration by proteolysis of vascular endothelial cadherin. Circulation research 102: 1192–1201. 18420943 10.1161/CIRCRESAHA.107.169805 PMC2818019

[19] Braun P, de Groot A, Bitter W, Tommassen J (1998) Secretion of elastinolytic enzymes and their propeptides by Pseudomonas aeruginosa. Journal of bacteriology 180: 3467–3469. 9642203 10.1128/jb.180.13.3467-3469.1998 PMC107305

[20] Hermant B, Bibert S, Concord E, Dublet B, Weidenhaupt M, et al (2003) Identification of proteases involved in the proteolysis of vascular endothelium cadherin during neutrophil transmigration. The Journal of biological chemistry 278: 14002–14012. 12584200 10.1074/jbc.M300351200

[21] Toussaint B, Delic-Attree I, Vignais PM (1993) Pseudomonas aeruginosa contains an IHF-like protein that binds to the algD promoter. Biochemical and biophysical research communications 196: 416–421. 8216322 10.1006/bbrc.1993.2265

[22] Breviario F, Caveda L, Corada M, Martin-Padura I, Navarro P, et al (1995) Functional properties of human vascular endothelial cadherin (7B4/cadherin-5), an endothelium-specific cadherin. Arteriosclerosis, thrombosis, and vascular biology 15: 1229–1239. 10.1161/01.atv.15.8.1229 7627717

[23] Corada M, Liao F, Lindgren M, Lampugnani MG, Breviario F, et al (2001) Monoclonal antibodies directed to different regions of vascular endothelial cadherin extracellular domain affect adhesion and clustering of the protein and modulate endothelial permeability. Blood 97: 1679–1684. 11238107 10.1182/blood.v97.6.1679

[24] Corada M, Zanetta L, Orsenigo F, Breviario F, Lampugnani MG, et al (2002) A monoclonal antibody to vascular endothelial-cadherin inhibits tumor angiogenesis without side effects on endothelial permeability. Blood 100: 905–911. 12130501 10.1182/blood.v100.3.905

[25] Prasain N, Stevens T (2009) The actin cytoskeleton in endothelial cell phenotypes. Microvascular research 77: 53–63. 19028505 10.1016/j.mvr.2008.09.012 PMC2700738

[26] Tamura Y, Suzuki S, Sawada T (1992) Role of elastase as a virulence factor in experimental Pseudomonas aeruginosa infection in mice. Microbial pathogenesis 12: 237–244. 1614334 10.1016/0882-4010(92)90058-v

[27] Wretlind B, Bjorklind A, Pavlovskis OR (1987) Role of exotoxin A and elastase in the pathogenicity of Pseudomonas aeruginosa strain PAO experimental mouse burn infection. Microbial pathogenesis 2: 397–404. 3148812 10.1016/0882-4010(87)90046-5

[28] Gavard J (2009) Breaking the VE-cadherin bonds. FEBS letters 583: 1–6. 19059243 10.1016/j.febslet.2008.11.032

[29] Azghani AO (1996) Pseudomonas aeruginosa and epithelial permeability: role of virulence factors elastase and exotoxin A. American journal of respiratory cell and molecular biology 15: 132–140. 8679217 10.1165/ajrcmb.15.1.8679217

[30] Vikstrom E, Tafazoli F, Magnusson KE (2006) Pseudomonas aeruginosa quorum sensing molecule N-(3 oxododecanoyl)-l-homoserine lactone disrupts epithelial barrier integrity of Caco-2 cells. FEBS letters 580: 6921–6928. 17157842 10.1016/j.febslet.2006.11.057

[31] Vikstrom E, Bui L, Konradsson P, Magnusson KE (2009) The junctional integrity of epithelial cells is modulated by Pseudomonas aeruginosa quorum sensing molecule through phosphorylation-dependent mechanisms. Experimental cell research 315: 313–326. 19038248 10.1016/j.yexcr.2008.10.044

[32] Schmidtchen A, Holst E, Tapper H, Bjorck L (2003) Elastase-producing Pseudomonas aeruginosa degrade plasma proteins and extracellular products of human skin and fibroblasts, and inhibit fibroblast growth. Microbial pathogenesis 34: 47–55. 12620384 10.1016/s0882-4010(02)00197-3

[33] Hong YQ, Ghebrehiwet B (1992) Effect of Pseudomonas aeruginosa elastase and alkaline protease on serum complement and isolated components C1q and C3. Clinical immunology and immunopathology 62: 133–138. 1730152 10.1016/0090-1229(92)90065-v

[34] Kuang Z, Hao Y, Walling BE, Jeffries JL, Ohman DE, et al (2011) Pseudomonas aeruginosa elastase provides an escape from phagocytosis by degrading the pulmonary surfactant protein-A. PloS one 6: e27091. 22069491 10.1371/journal.pone.0027091 PMC3206073

[35] Mariencheck WI, Alcorn JF, Palmer SM, Wright JR (2003) Pseudomonas aeruginosa elastase degrades surfactant proteins A and D. American journal of respiratory cell and molecular biology 28: 528–537. 12654643 10.1165/rcmb.2002-0141OC

[36] Beaufort N, Corvazier E, Hervieu A, Choqueux C, Dussiot M, et al (2011) The thermolysin-like metalloproteinase and virulence factor LasB from pathogenic Pseudomonas aeruginosa induces anoikis of human vascular cells. Cellular microbiology 13: 1149–1167. 21501369 10.1111/j.1462-5822.2011.01606.x

[37] Dulon S, Leduc D, Cottrell GS, D'Alayer J, Hansen KK, et al (2005) Pseudomonas aeruginosa elastase disables proteinase-activated receptor 2 in respiratory epithelial cells. American journal of respiratory cell and molecular biology 32: 411–419. 15705968 10.1165/rcmb.2004-0274OC

[38] Leduc D, Beaufort N, de Bentzmann S, Rousselle JC, Namane A, et al (2007) The Pseudomonas aeruginosa LasB metalloproteinase regulates the human urokinase-type plasminogen activator receptor through domain-specific endoproteolysis. Infection and immunity 75: 3848–3858. 17517866 10.1128/IAI.00015-07 PMC1951998

[39] Durand E, Michel G, Voulhoux R, Kurner J, Bernadac A, et al (2005) XcpX controls biogenesis of the Pseudomonas aeruginosa XcpT-containing pseudopilus. The Journal of biological chemistry 280: 31378–31389. 16012171 10.1074/jbc.M505812200

[40] McIver KS, Kessler E, Olson JC, Ohman DE (1995) The elastase propeptide functions as an intramolecular chaperone required for elastase activity and secretion in Pseudomonas aeruginosa. Molecular microbiology 18: 877–889. 8825092 10.1111/j.1365-2958.1995.18050877.x

[41] Cisz M, Lee PC, Rietsch A (2008) ExoS controls the cell contact-mediated switch to effector secretion in Pseudomonas aeruginosa. Journal of bacteriology 190: 2726–2738. 18039770 10.1128/JB.01553-07 PMC2293250

[42] Pastor A, Chabert J, Louwagie M, Garin J, Attree I (2005) PscF is a major component of the Pseudomonas aeruginosa type III secretion needle. FEMS microbiology letters 253: 95–101. 16239085 10.1016/j.femsle.2005.09.028

