# Supplementary figures and images for: VE-Cadherin Cleavage by LasB Protease from Pseudomonas aeruginosa Facilitates Type III Secretion System Toxicity in Endothelial Cells
Source: PLoS Pathog. 2014 Mar 13;10(3):e1003939. doi: 10.1371/journal.ppat.1003939 (PMC3953407; doi:10.1371/journal.ppat.1003939)

**A**

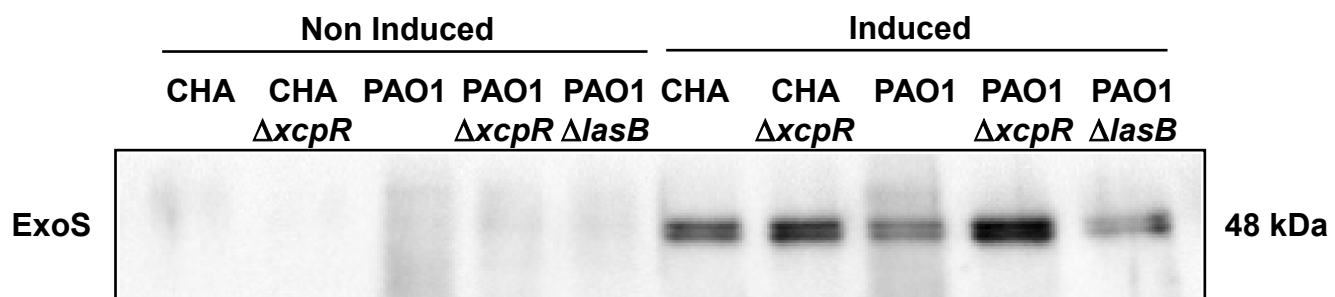

**B**

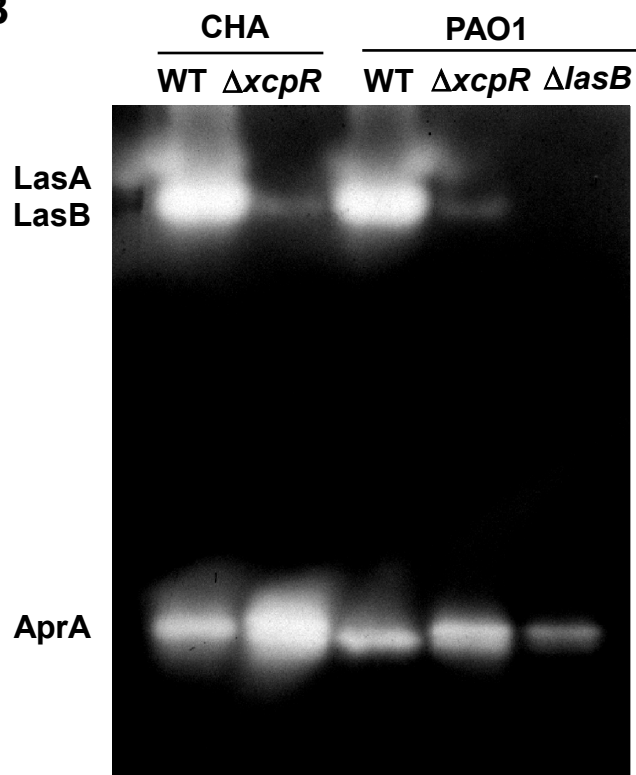

Supplement: Figure S1 — Type 2 and type 3 secretions of P. aeruginosa strains. (A) Secretomes (at A600 = 1.0) were produced in LB (Non induced) or in LB supplemented with 5 mM EGTA to induce T3 effector secretion (Induced). The secretomes (16 µL) were analyzed by Western blot using ExoS antibodies. ExoS was secreted by all analyzed strains in induced conditions. (B) Secretomes (10 µL) collected at A600 = 1.5 were analyzed by electrophoresis in gelatin-containing SDS-PAGE. After overnight incubation, in-gel protein degradation was assayed by coomassie staining. Protease electrophoretic pattern was as previously reported [16]. LasA and LasB activities were minimal in ΔxcpR strains, but also in the LasB-deficient strain because of the required LasB activation of LasA [19]. Remaining LasA/LasB activities observed in ΔxcpR strains probably originate from lysed bacteria. Activity of AprA, a T1SS protease, was present in all secretomes. (PDF) [file ppat.1003939.s001.pdf]

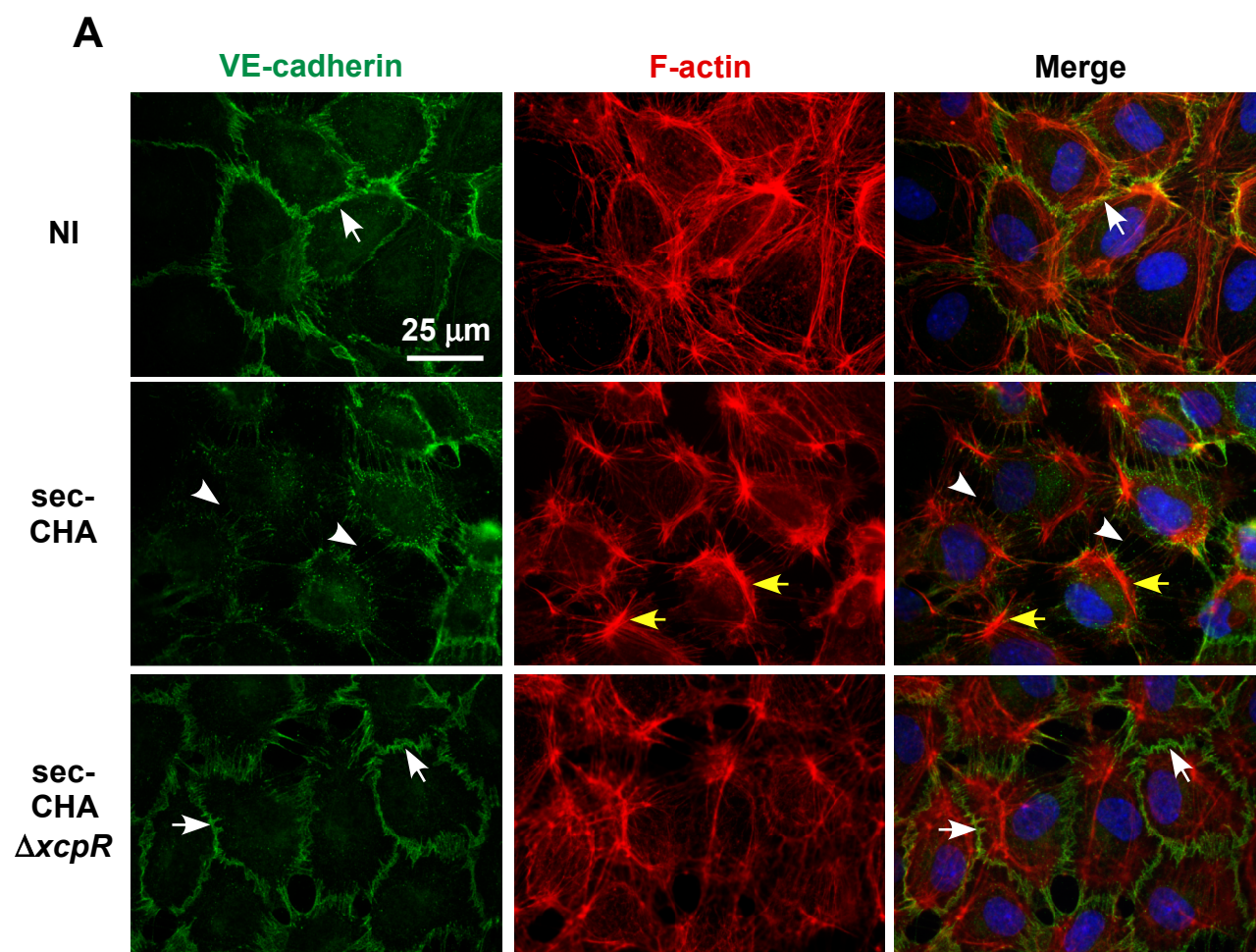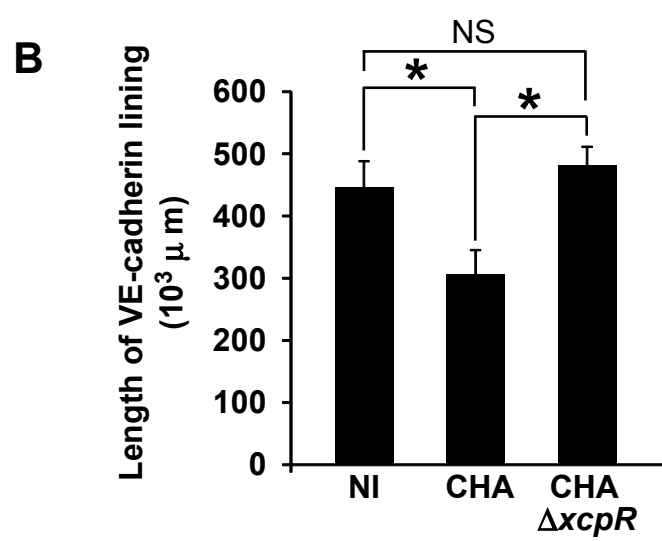

Supplement: Figure S2 — P. aeruginosa 's type 2 effectors provoke gap formation in endothelial cell monolayer. (A) Confluent HUVECs were treated with a 10% volume of CHA or CHAΔxcpR secretomes (sec) or LB in the cell culture medium. Cells were fixed 1.5 hours later and labeled for VE-cadherin (green) and filamentous (F)-actin (red). In the merged images, nuclei are also shown in blue. The actin cytoskeleton is present in all 3 conditions, however, as for PAO1, there were fewer actin fibers and the onset of condensed cortical actin foci (yellow arrows) in retracted cells (sec-CHA). The VE-cadherin antibody decorates cell-cell junctions in LB or in sec-CHAΔxcpR conditions (arrows), while labeling is attenuated or absent in presence of sec-CHA (arrowheads). This alteration parallels gap formation between cells. A similar experiment was performed with PAO1 secretomes with identical results (Fig. 2). (B) Length of VE-cadherin lining at cell edges was quantified on 10 images for each condition. Data represent the mean+SD of VE-cadherin lining length. Statistics: 1-way ANOVA, p<0.001; Significance was determined using pairwise Bonferroni's test (*). (PDF) [file ppat.1003939.s002.pdf]

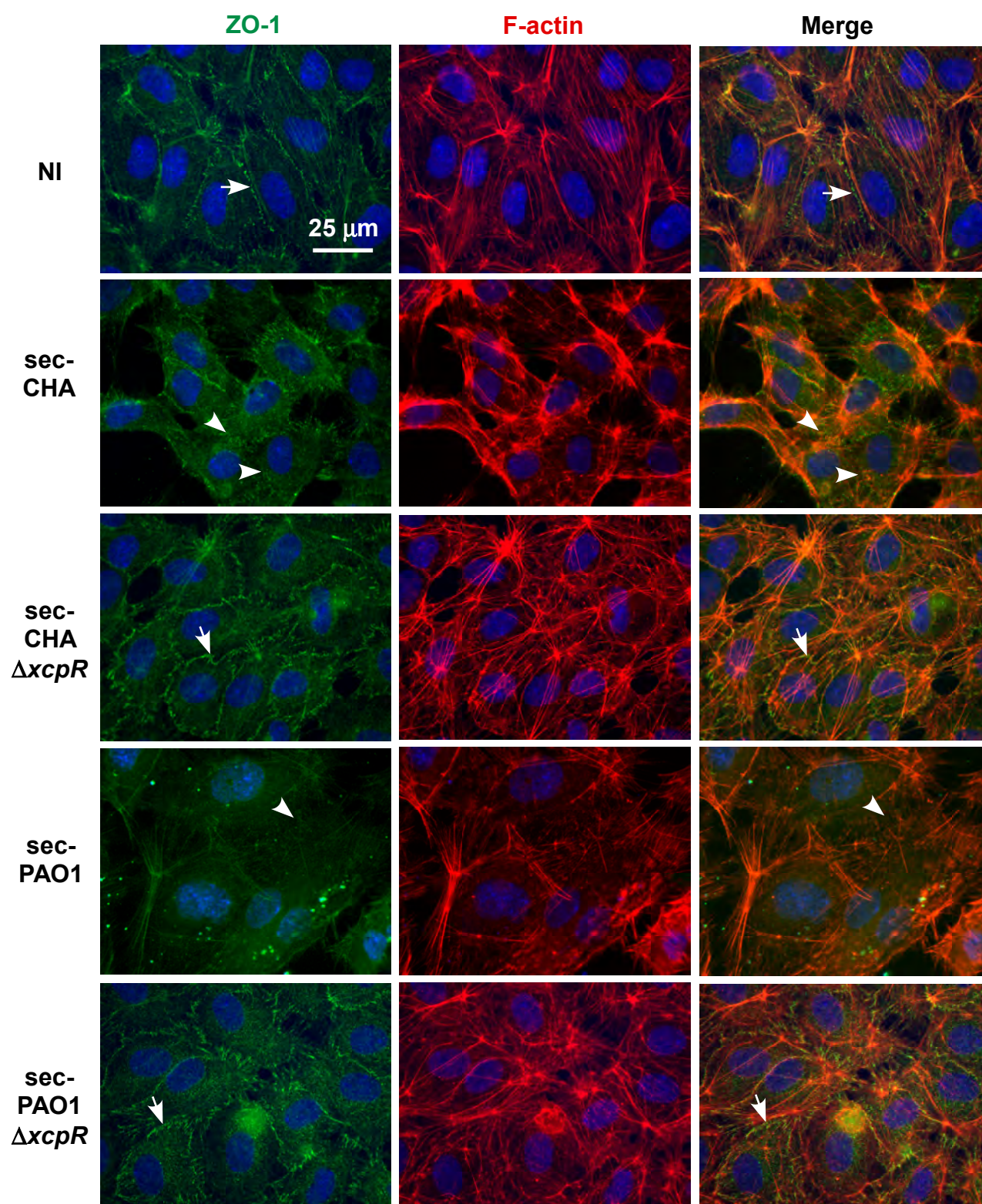

Supplement: Figure S3 — Secretomes from P. aeruginosa disrupt tight junctions. Confluent HUVECs were treated for 3 hours with PAO1 or CHA secretomes either wild type or ΔxcpR as indicated. Controls were noninfected cells (NI). Cells were fixed and labeled for F-actin (red) or ZO-1 (green), an essential tight junction component. ZO-1 antibody produced a very thin staining at cell-cell junctions (arrows); in presence of secretomes from wild-type strains, labeling disappeared (arrowheads). (PDF) [file ppat.1003939.s003.pdf]

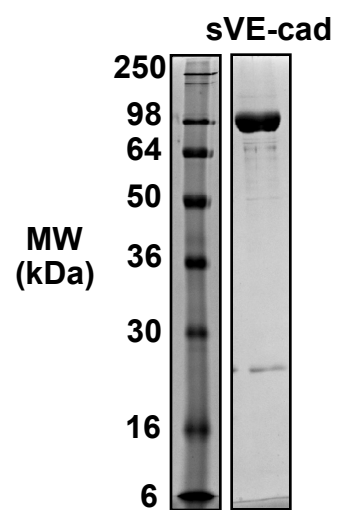

Supplement: Figure S4 — Electrophoretic analysis of purified soluble VE-cadherin. VE-cadherin extracellular domain fused to 6-histidine tag (sVE-cad) was produced in HEK-293 cells. Recombinant protein was purified from conditioned medium by ion exchange and nickel-histidine affinity chromatographies successively. Purified protein was electrophoresed and gel was Coomassie stained. Data shows a major band at 90 kDa and a very faint band at 25 kDa. (PDF) [file ppat.1003939.s004.pdf]

**LasB**

**MW**

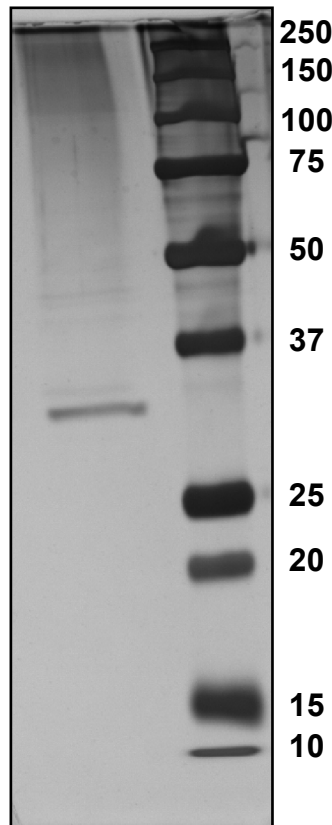

Supplement: Figure S5 — Electrophoretic analysis of purified LasB. Electrophoretic analysis and silver staining of purified LasB shows one band at 35 kDa. (PDF) [file ppat.1003939.s005.pdf]

PAO1

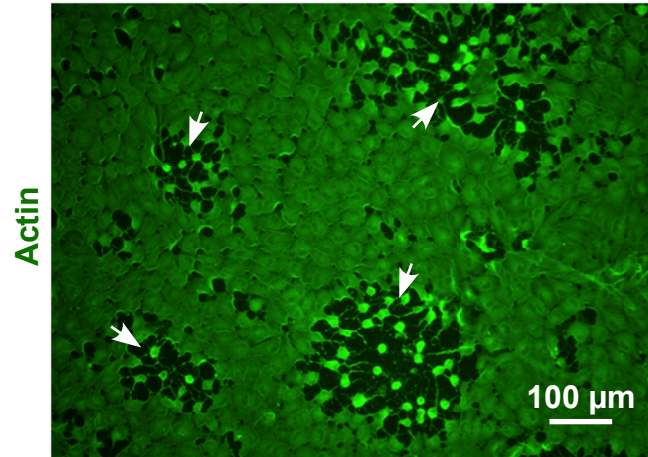

Supplement: Figure S6 — Propagation of cell retraction in HUVEC monolayer. Confluent HUVECs were infected for 2 hours with PAO1 at MOI = 10. Cells were fixed and labeled with actin antibody (green). Endothelial cell retraction was not synchronized, but started in specific points and propagated from these sites (arrows). (PDF) [file ppat.1003939.s006.pdf]
